# Regional organisations supporting health sector responses to climate change in Southeast Asia

**DOI:** 10.1186/s12992-018-0388-z

**Published:** 2018-08-03

**Authors:** Daniel Gilfillan

**Affiliations:** Fenner School of Environment and Society, The Australian National University, 48 Linnaeus Way, Acton, ACT, 2601 Australia

**Keywords:** Southeast Asia, Health, Adaptation, Governance, Climate change, Coordination

## Abstract

**Background:**

The role played by regional organisations in climate change adaptation and health is growing in Southeast Asia, with the Asian Development Bank and the Asia-Pacific Regional Forum on Health and Environment both supporting health and adaptation initiatives. There is, however, a lack of empirical research on the value that regional organisations add to national health-related adaptation. This qualitative research compares regional project and governance-based models of adaptation and health support in Southeast Asia, providing an analysis of strengths and weaknesses of each, as well as possibilities for improvement.

**Methods:**

An existing adaptation assessment framework was modified for this research, and used as a guide to gather and analyse data from academic and grey literature, policy documents and interviews in order to qualitatively assess two organisations and their different models of adaptation and health support.

**Results:**

This research found differing strengths in the approaches to climate change and health used by the Asian Development Bank and by the Asia-Pacific Regional Forum on Health and Environment. The regional forum has vision, high levels of perceived legitimacy, and access to ‘in-house’ expertise in public health and climate change. Conversely, the Asian Development Bank has strengths in project management and access to significant financial resources to support work in climate change and health.

**Conclusion:**

When regional organisations, such as the Asian Development Bank and the Asia-Pacific Regional Forum on Health and Environment, have membership and mandate overlaps, their work will likely benefit from well designed, institutionalised and incentivised coordination mechanisms. Coordination can reduce redundancies as well as the administrative workload on partner government agencies. In the case-study examined, the Asian Development Bank’s project management expertise complements the vision and high levels of perceived legitimacy of the Asia-Pacific Regional Forum on Health and Environment, thus a coordinated approach could deliver improved adaptation and health outcomes.

**Electronic supplementary material:**

The online version of this article (10.1186/s12992-018-0388-z) contains supplementary material, which is available to authorized users.

## Introduction

Southeast Asia comprises 11 countries, ranging from archipelagos and an island-state, to landlocked Laos. Despite their varied geographies, developing countries in Southeast Asia have broadly similar climate change-related vulnerabilities due to a high and growing population and a reliance on agriculture for livelihoods [1]. The Intergovernmental Panel on Climate Change (IPCC) [2] defines vulnerability as a function of several elements, including physical impact exposure (e.g. changing rainfall patterns) and adaptive capacity (e.g. ability to adjust one’s means of livelihood support). For example, Yusuf and Francisco [3] rated Cambodia’s climate change vulnerability as high because, despite relative insulation from physical climate change impacts, the country’s poor infrastructure, widespread poverty and lack of technology means it has low adaptive capacity. Similarly, McElwee [4] described the high vulnerability levels of the many people of the Mekong Delta whose poverty and poor education limit their capacity to maintain livelihoods in the face of climate-related shocks including rising sea-level induced salt-water intrusion into agricultural areas. Broadly similar regional vulnerabilities highlight the importance of regional organisations (ROs) supporting climate change adaptation (CCA) in Southeast Asia. Regional organisations are supra-national institutions with national governments as members, established to address shared problems and focussing their activities in a specific region.

According to the IPCC, CCA is the “process of adjustment to actual or expected climate and its effects” ([2], p. 1758). For health, this includes more than climate-proofing treatment centres and preparing for more cases of climate-sensitive diseases, because health determinants include factors both inside and outside the direct control of health ministries, including “access to safe water and clean air, food security, strong and accessible health systems, and reductions in social and economic inequity” ([5], p. 2). Missoni [6] argued that inequality, including health inequality, is exacerbated by a focus on economic growth and, further, that addressing health determinants ahead of time is less expensive and leads to longer-term sustainability. Similarly, improving health determinant access will increase adaptive capacities, emphasising the growing importance of interactions between health and CCA [7, 8]. However, addressing health determinants ahead of time requires cooperation and coordination across different sectors, scales, and across different administrative regions (e.g. [9]). Following Gilfillan et al. [9], coordination in this paper refers to both functional cooperation, where tasks are distributed between actors, and to collaborative cooperation, where actors work collaboratively towards agreed goals.

Environmental health determinants have attracted the interest of governments across Southeast Asia. For example, 14 health and environment ministries worked with the World Health Organization (WHO) and United Nations Environment Programme (UNEP) to establish the Regional Forum on Environment and Health [10], now the Asia-Pacific Regional Forum on Health and Environment (APRF), which has climate change and health objectives. The APRF, with its intergovernmental meeting of 34 member states across the Asia-Pacific region, secretariat, and seven thematic working groups (TWGs), meets the RO definition described above. The Asian Development Bank (ADB), an RO with 67 member states from within and outside the Asia-Pacific Region, is also focussing increasing amounts of time and energy on transnational environmental challenges [11, 12], with its 2008–2020 strategic plan including climate change impacts on health as a concern [13].

The purpose of this paper is to examine the effectiveness of ROs supporting CCA and health in Southeast Asia, and to the best of the researcher’s knowledge, it is the first research to do so. Using a case study approach, and reflecting on other research into international and regional organisations, treaties and regimes (e.g. [14, 15]), this research concludes that collaborative cooperation could improve the effectiveness of the adaptation and health related work of ROs such as the ADB and APRF.

## Background

A brief background is provided here on climate change and health, organisational legitimacy and the involvement of ROs in addressing climate change impacts on health in Southeast Asia.

### Public health and climate change

The literature on climate change and health is evolving rapidly. For example, Hess et al. [16] reviewed the work of authors including Ebi and Burton [17], Ebi et al. [18], Ebi and Semenza [19], Ebi et al. [20], concluding that while there is substantial literature on identifying climate change impacts on public health, little focusses on public health interventions. To reconcile evidence-based public health (EBPH) with CCA and health, Hess et al. [16] modified Jones et al*’s* [21] EBPH framework to incorporate CCA concerns. Likewise, the United States Centers for Disease Control and Prevention developed the standalone ‘Building Resilience Against Climate Effects’ (BRACE) framework, which Marinucci et al. [22] described as suitable for assessing climate-related impacts on health as well as for prioritising interventions. While these authors focussed on the climate-health interface, the research presented here focusses on how effectively ROs are supporting health related CCA.

### Organisational legitimacy

Organisational effectiveness can be affected by stakeholder judgements about its legitimacy, or right, to act on behalf of stakeholders. Achieving goals is one way of building legitimacy, with Biermann and Gupta [23] describing output legitimacy, derived from successfully achieved objectives, being distinct from process-related input legitimacy. For example, Riggirozzi and Grugel [24] argued that the Union of South American Nations sought to create output-focussed legitimacy for itself by supporting health policy development across South America. Conversely input legitimacy is often associated with stakeholder inclusion in decision-making processes, and is perspective-dependent [23]. For example, an excluded group would likely give a lower process legitimacy rating than would beneficiaries of that exclusion. Linking input and output legitimacy, Kapiriri [25] argued that, for priority setting in low income countries, clear and transparent processes increase perceptions of legitimacy, thus leading to better stakeholder agreement. Similarly, Bernstein [26] argued that legitimacy is a key source of authority in global environmental governance where coercion is unavailable and inducements are costly. Thus, despite legitimacy being perspective-laden, it can be a key to achieving goals, particularly where carrot and stick approaches are not viable.

### Health, adaptation and regional Organisations in Southeast Asia

Despite evidence of ROs focussing attention on climate change and health (see e.g. [10, 13]), literature focussing on ROs, CCA and health is scarce. In one paper, Thomson et al. [27] argued that there is a need for clear evidence of climate and health links, and also a need for evidence that responses will be cost-effective. Supporting IPCC and WHO assessments, Wahlqvist et al. [28] argued that food security will be a major CCA and health issue in Asia, however none of the three regional initiatives they researched prioritised health.

Southeast Asia’s “ASEAN way” enshrines a concept of non-interference in other nations domestic affairs [12]. Despite this, authors including Brömmelhörster [29] and Koh and Bhullar [12] have discussed the importance of national adaptation strategies in Southeast Asia, supported by supra-national regional coordination, collaboration and knowledge sharing. Globally, supra-national climate-related cooperation relies on national rather than international legislation [30, 31], and thus, even where regional legislation is unlikely to eventuate, such as in Southeast Asia, ROs can support national CCA and health efforts. This could be done by, for example, supporting national efforts to develop climate change and health related evidence, and through catalysing domestic policy and legislation development.

### Theoretical framework

This research qualitatively assesses ROs supporting CCA and health, and is built on modern organisation theory, with the researcher also influenced by resilience thinking. Resilience thinking links closely to sustainability ideals. It acknowledges a dynamic external environment that requires individuals and systems to continuously and iteratively adapt [32, 33]. Modern organisation theory recognises the importance of different scales working together, with individuals working within broader systems and processes. It also recognises the importance of decision-making and communication processes and how these link with organisational goals [34]. Scott ([35], p. 16) argued that identifying the “mutual dependency” between principle parts of the organisation is a key to understanding organisational dynamics. Adding resilience thinking supports explicit recognition of factors external to the organisation, such as climate change-related health impacts and other organisations. It also means recognising that changes are occurring, and that uncertainty is not an excuse for inaction [see for example Hallegatte’s [36] description of strategies to cope with uncertainties associated with climate change]. It further recognises that system changes are possible without losing organisational identity [see for example Jacque’s [37] description of Japan’s response to forced engagement with the West in the 1800s]. Resilience thinking and modern organisation theory both complement the researcher’s view that sustainability will be enhanced when ROs, and their support for CCA and health, are regularly reviewed to reflect both environmental and institutional changes at global and national levels.

## Methods

### Analytical framework

Conceptually, organisational effectiveness is about an organisation’s systems, procedures and resources providing it with the capacity to achieve outputs and outcomes, as well as about its actual success in achieving those goals. For example Scott [35] premised effectiveness on goal achievement as well as resource acquisition ability, internal processes and stakeholder satisfaction. Despite its conceptual simplicity, there is a lack of agreement on suitable organisational effectiveness measures [38]. Including CCA and public health in organisational goals increases the challenge because both are future-focussed. Robinson and Gilfillan [15] reviewed the organisational effectiveness literature for CCA-related effectiveness indicators, developing the Framework for Assessing Regional Organisations Coordinating Climate Change Adaptation (FAROCCCA) because of a lack of suitable existing assessment frameworks. FAROCCCA, as used for this research, is a tool comprising 63 specific indicators of organisational effectiveness relevant to CCA and health. The 63 indicators were developed by Robinson and Gilfillan [15] based on previous work on organisational effectiveness across a variety of disciplines and literatures, such as private and public sector effectiveness [39], resource management (e.g. [40]), and modern organisational theory (e.g. [41]). FAROCCCA includes elements of four main models of organisational effectiveness: 1) the goal oriented model, which focusses on immediate organisational outputs [42], 2) the resource-oriented model, which is premised on an organisational need to acquire resources in order to function [43], 3) the process-oriented model, which considers how effectively those resources are used [41], and 4) the strategic constituency model, which concerns itself with relationships and linkages between an organisation and its primary stakeholders [44]. The evidence gathered against a single FAROCCCA indicator provides one data point, but using the framework to systematically compile and analyse evidence can provide useful insights into organisational operations.

The 63 FAROCCCA indicators are split between 18 sub-components, ranging from goals and governance to initiative design logic and collaboration, as shown in Table 1 in the results section. The sub-components are further split between the following three FAROCCCA components:

1. The organisation’s inputs, which are the characteristics that provide the organisations with its capacity to undertake adaptation- and health-related work. Following Sowa et al. [45], the premise for assessing organisation inputs is that a well-managed and structured organisation has a higher chance of delivering quality initiatives and hence achieving its goals.
2. The organisation’s initiatives. Initiatives are projects and other mechanisms by which organisations work towards their goals. For example, the ADB pursues its goal of assisting member countries to adapt to health-related climate change impacts through its Strengthening Resilience to Climate Change in the Health Sector in the Greater Mekong Subregion (SRCC) project.
3. The organisation’s outputs. Outputs are the immediate results of an initiative, and are distinct from longer-term outcomes. For example, outputs include training courses and awareness raising sessions, but do not refer to intended or unintended behaviour changes that result from these.

For the Southeast Asian CCA and health context, FAROCCCA required some modifications including ensuring indicators referred to CCA and health where appropriate, and allowing for an intergovernmental meeting, its TWGs and secretariat to be assessed as an organisation. A third modification was the inclusion of an explicit legitimacy indicator, with the addition supported by the work of authors such as Kapiriri [25], Bernstein [26] and Riggirozzi and Grugel [24]. Organisational legitimacy, as these authors outline, is an external assessment of the organisation. In contrast, rating ROs against some FAROCCCA indicators requires the candid judgements of those with intimate knowledge of the RO’s internal dynamics. These are referred to as perceptual indicators (see e.g. [45]). An example of a perceptual indicator is indicator 7 in sub-component 2 of component 1 of FAROCCCA: “*Leaders create a dynamic organisational/forum/project culture, making it a desirable place to work”.*

Table 1, in the results section, provides a summary of the modified FAROCCCA results, grouped into the 18 FAROCCCA sub-components. Additional file 1 shows the modifications to the original FAROCCCA indicators, along with indicator results. Additional files 2 and 3 provide indicator detail as well as the results with evidence for the two organisations. Along with results and evidence, a rationale for each rating is given, italicised and in bold.

### Research methods

This research builds on national level work on climate change adaptation and health governance (e.g. [9, 46, 47, 48]), and qualitatively assesses two distinct models of regional support for CCA and health; 1.) the ADB’s project-based model, and 2.) the APRF’s governance model. Initial data collection consisted of 22 in-depth, semi-structured interviews, which were conducted over an 18 month period between September 2015 and February 2017. A 14 question interview guide was approved under the ethics approval for this research, and this was used to structure interviews. Interviews were conducted in English and ranged in length from 25 min to 105 min, with an average length of 52 min. Nineteen interviewees were male, and 3 were female, indicating that regional level governance of climate change and health is a male dominated area. Interviewees represented 11 organisations at national and regional levels, as well as representing three countries in addition to the ROs. Interviewees were given a participant information sheet detailing the research objectives and a number to contact for questions or concerns. Interviewees provided the researcher with either written or recorded consent for the interview to be recorded and used for this research, and were advised that they need only answer questions they were comfortable with. Transcripts were provided to interviewees for their comment and approval. Following this, using FAROCCCA as an analytical tool, interview data was triangulated against an analysis of policy documents, academic publications and grey literature such as annual and financial reports, evaluation documents and strategic plans. Document analysis and interviews focussed on current directions and trends, however it also included reference to charter documents dating back to 1994 and 2007 for the ADB and APRF respectively. The most recent document analysed was published in 2018.

Interviewees were selected for their direct experience and expertise, and their ability to comment on the ADB and APRF initiatives. They were all senior personnel with oversight of climate change and/or health portfolios, with well-grounded understandings of policy, climate change and health interactions. Respondents came from national health and environment ministries as well as from the ADB and APRF, or were expert environment and health observers with close links to the ADB and APRF.

Research data was reviewed to gather evidence against each of the modified FAROCCCA indicators. Each indicator was rated, following the ‘traffic light method’ and qualitative assessment protocols [40]. The ratings were used principally as a means to highlight points of interest and comparisons between the organisations and their models of support. Each rating reflected the indicator wording. For example, where an indicator specified ‘evidence of’, lack of evidence resulted in a negative rating, but where an indicator specified a particular characteristic, lack of evidence of this resulted in a ‘no evidence’ rating.

This research had some limitations. First, despite the importance of understanding whether health and adaptation initiatives are achieving sustainable long-term outcomes, the ongoing nature of the initiatives precludes their measurement, as per Mitchell [49]. While many health interventions into infectious disease control, for example, are measured during implementation, assessing adaptation and health interventions is a special case. This is both because it is only recently that public health focussed responses to climate change have begun to be implemented, and because adapting to climate change means adapting to a situation that does not yet exist. For both these reasons there is a paucity of empirical evidence about the long-term outcomes of adaptation and health interventions. Thus the research relied on evidence of whether the assessed initiatives are on track to achieve their immediate adaptation and health outputs. A second limitation related to the inclusion of perceptual indicators. These would be useful, for example, for the organisations to undertake self-assessments, but were outside the scope of this research.

## Results

A narrative of salient results is presented here following the three components of FAROCCA: organisational inputs, initiatives, and outputs. A summary of the full results is presented below in Table 1, with Additional files 2 and 3 detailing evidence against each indicator for both organisations. Interview data is cited using interviewee numbers. For example, interviewee number 6 is cited as [#06].

**Table 1:** Results Summary for Two Models of Regional Support for National Level Adaptation and Health

*Component one: Input effectiveness (35 indicators)*												
	APRF						ADB					
Sub-component	☑	☒	◈	(NE)	(PI)	# of indicators	☑	☒	◈	(NE)	(PI)	# of indicators
1. Goals	3	1	–	–	–	4	1	3	–	–	–	4
2. Governance and leadership	4	–	2	–	2	8	4	1	1	–	2	8
3. Resources	4	3	2	1	1	11	8	1	1	–	1	11
4. Structure, systems and processes	–	–	1	–	7	8	1	–	–	–	7	8
5. Research and collaboration capacity	4	–	–	–	–	4	4	–	–	–	–	4
*Component two: Project/initiative effectiveness (20 indicators)*												
	CCTWG						SRCC project					
Sub-component	☑	☒	◈	(NE)	(PI)	# of indicators	☑	☒	◈	(NE)	(PI)	# of indicators
1. Needs and goals	4	1	1	–	–	6	5	–	1	–	–	6
2. Scope	2	–	–	–	–	2	2	–	–	–	–	2
3. Logic, design and adequacy	1	1	–	–	–	2	2	–	–	–	–	2
4. Resources	1	2	–	2	–	5	4	–	1	–	–	5
5. Technical efficiency	1	–	–	–	–	1	–	–	1	–	–	1
6. Implementation	–	–	1	–	–	1	–	1	–	–	–	1
7. Monitoring and evaluation	–	2	–	–	–	2	–	1	1	–	–	2
8. Sustainability	–	–	–	1	–	1	–	–	–	1	–	1
*Component three: Output effectiveness (8 indicators)*												
	APRF						ADB					
Sub-component	☑	☒	◈	(NE)	(PI)	# of indicators	☑	☒	◈	(NE)	(PI)	# of indicators
1. Goal attainment	1	–	–	–	–	1	1	–	–	–	–	1
2. Research and knowledge management	2	–	–	–	–	2	2	–	–	–	–	2
1. Collaboration and advocacy	2	–	–	–	–	2	2	–	–	–	–	2
4. Education and training	2	–	–	–	–	2	2	–	–	–	–	2
5. Specialised advice provided	1	–	–	–	–	1	1	–	–	–	–	1

RATING SYSTEM

☒ No

◈ To some extent

☑ Yes

*NE* No evidence

*PI* Perceptual measure or measure not rated in this paper

### Inputs

This research examines two models of support for CCA and health. The ADB primarily provides support through grant-based projects for health sector CCA. In contrast the APRF uses peer-pressure to encourage national ownership of environment and health. As shown in Table 1, the ADB and APRF rate similarly for governance and leadership and collaboration capacity, while the ADB has significantly better resourcing than the APRF.

Both organisations have mandates supporting CCA and health initiatives, though this was not always the case. According to the APRF charter, “[g]overnments [at all levels] should address the health impacts and implications of […] priority areas of environmental concern” including “[c]limate change” [10, Article 3]. In contrast, the ADB Charter specifies that “[o]nly economic considerations shall be relevant to” decisions of “[t]he Bank, its President, Vice-President(s), officers and staff” ([50], Article 36(2)). However, 14 years later, the ADB’s 23 page 2008–2020 strategic plan included three quarters of a page on environment, and committed to support developing member countries adaptation “to the unavoidable impacts of climate change—including those related to health” ([13], p. 14). This is in line with the growing global recognition of the importance of climate change and health concerns [7], and highlights the mandate convergence between the ADB and APRF [10, 13].

APRF members are health and environment ministries, with resources and responsibilities as allocated by their governments. While recognising Southeast Asian developing country resource constraints [#02], the APRF pursues the vision of encouraging national ownership of environment and health concerns, such as erratic rainfall resulting in food insecurity [#19], by finding a,

“mechanism where they [member states] have to do [environment and health-related work]. So for example, like we inviting them for a regional meeting, to update – is a kind of informal subtle way for them [to have the encouragement] to do the [environmental health] country profile [#06].”

This subtle encouragement appears to be having some success, “from the [last] high level meeting […] WHO and UNEP asked […] members to develop or update the environmental [health] country profile, and then send back […] – we already send” [#10, similarly #11].

Likewise, commenting on the APRF initiated National Environmental Health Action Plan (NEHAP) initiative, a senior Vietnamese government official reported that “I think in the future our government will support NEHAP [but] I think we have to do a lot of things to change […] our government[‘s] idea[s]” [#07]. This comment also reflects the current low prioritisation of environment and health at the national level, with the NEHAP recently rejected at the ministerial level because of disagreements about which ministry should be responsible [#05]. Vietnam’s rejection of the NEHAP highlights that even though some environment and health related project work is authorised by the government, there is a lack of interest in creating environment and health legislation. Another example is the environment ministry recently prioritising the facilitation of investment in large development projects over integration of health impact assessment criteria into its environmental impact assessment (pers. comm., May 2018). These issues in Vietnam are mirrored in Cambodia, where CCA and health is only prioritised when there is international funding available [#09], with international funding limited to project timelines [48]. These examples from Vietnam and Cambodia highlight the need for national CCA and health ownership, so that these issues can be addressed in the long-term. This is because, while a multi-lateral development institution such as the ADB can stipulate that projects that it implements comply with global environment and health norms [51], national governments are responsible for regulating a substantially larger share of national expenditure. For example, in Myanmar, internationally sourced development loans and grants amount to around 7% of national government expenditure [52], with these figures not including private sector investment. Thus, the APRF goal of building national ownership is worth pursuing, and the success it has achieved so far indicates that its methods are worth considering.

In contrast to the APRF, the ADB provides resourcing for environment and health work it supports. For example, in June 2015 the ADB announced a $4.4 million grant for its Strengthening Resilience to Climate Change in the Health Sector in the Greater Mekong Subregion (SRCC) project [53]. Despite the funding, the ADB faces issues with “soft component[s] of the health sector project[s]” because national governments “love to borrow for mortar and equipment and bricks, but they are very reluctant in investing in capacity development”, and grant finance for the soft components is limited [#16]. Another ADB limitation is in-house technical capacity, thus experienced external consultants are engaged for particular projects. For example, the consultants managing the SRCC project include authors of multiple academic climate change and health publications focussing on South East Asia (see e.g. [46, 54]). While the ADB facilitates resourcing for CCA and health projects, the APRF goal is for national ministries to take ownership of climate change and health related issues.

### Initiatives

The APRF’s climate change thematic working group (CCTWG) is a forum for senior health and environment ministry personnel from countries in the region to meet, discuss and provide mutual support in addressing common climate change and health-related concerns. The ADB’s SRCC project supports three Greater Mekong Sub-region (GMS) countries to reduce the vulnerability of their poor and marginalised populations to climate-related health risks, and is being implemented by a consultancy firm [55]. Overall the effectiveness of the ADB’s SRCC project rates more positively than the CCTWG initiative, with 13 positive and 2 negative ratings out of 20 compared with the CCTWG’s 9 positive and 6 negative (see component 2 in Table 1). This positive rating for its SRCC project is linked to other indicators of the ADB’s strength in project management. For example, the ADB describes itself as having a comparative strength in infrastructure project management [56], and it has demonstrated project success supporting development of environmental impact assessment (EIA) procedures for China, so that China now has “one of the best EIA systems in the world” [#04].

The two initiatives have some similarities. First, both include a capacity building focus. The SRCC project seeks to enhance the “capacity of participating countries and health agencies in climate change adaptation” ([55], Online), and the APRF’s CCTWG focusses on capacity building and regional knowledge management [57]. Second, in line with academic perspectives (e.g. [27, 58]), both organisations recognise the importance of quantitative evidence to build momentum on climate change and health concerns. The ADB’s SRCC project recommended the development of future plans “that include health impacts and evidence-based good practices on climate change and health for key sectors” ([55], Online), and the APRF has produced a 55-page synthesis report of members’ environment and health country profiles (EHCPs), including a five page chapter on climate change impacts [59]. Third, there is evidence that both initiatives fill an adaptation need. The CCTWG, because strengthening human resource capacity is considered as a priority issue for CCA and health (see e.g. [46, 60, 61]), and the ADB SRCC project documents include detailed information about climate change health impacts, including differentiation of causes between the three target countries.

Even though they are responding to adaptation needs, both initiatives face implementation issues, with the CCTWG challenges being larger than the SRCC project’s. The CCTWG is working to build bridges between government sectors, but as one respondent noted, “2013 or 2014 – it was the first and maybe the only working group on climate change – thematic working group on climate change. We [are] so much behind in that” [#06]. The results from Table 1 show poor project management of the CCTWG initiative. For example, there was a lack of specific and measurable objectives and an absence of monitoring and evaluation, without which, possibilities for utilising the APRF’s subtle encouragement approach are constrained.

While the SRCC project is performing better than the CCTWG it too has had implementation challenges. For example, the project inception workshop was run a year after the initiative was scheduled to begin, and there were also project design weaknesses. One weakness was that while relevant countries were consulted, authorship of project documents resided with the bank itself (e.g. [62]). This may be ideal from an expert knowledge perspective, but has left national government agencies feeling alienated, as if they are project conduits, rather than project partners (researcher’s observation, see also Kapiriri [25] for arguments about development partners and legitimacy of priority setting in low income countries). This alienation was exacerbated because contractual arrangements between the consulting firm and the ADB made international consultants responsible for drafting key project implementation documents (pers. comm., Feb 2017). These two factors led to a reduction in perceived project legitimacy as well as frustrations on the part of both government agencies and the implementing organisation. They are also likely a factor in the project having expended less than US$1.9 Million of the its US$4.4 Million budget by May 2018, with less than six months remaining on the project’s original three year timeline [55].

Another weakness is that the project aim of reducing climate change vulnerabilities of poor and marginalised populations is not clearly linked to the project’s objective of improving the coping capacity of governments to health-related climate change impacts. It is possible that some of these weaknesses relate to difficulties the ADB has with ‘soft’ project components [#16]. The discussion section of this research further analyses the weaknesses of the ADB’s and APRF’s CCA and health initiatives, and proposes a solution to improve them.

### Outputs

Both organisations’ outputs rated well. For example, the evidence gathered relating to the FAROCCCA sub-component on collaboration and advocacy shows that both organisations do work with stakeholders, although their methodologies differ. In this regard, the ADB has some limitations in collaborating with government agencies in the region. For example, “our Vietnamese partner, it is the first time we’ve worked with them, and they don’t understand the ADB procedures, and so because we work basically in one way – we’re giving you money, and you have to do it this way” [#13], with [#14] making similar remarks regarding Myanmar. Comparatively the APRF shows a greater collaborative focus. For example, there was a substantial amount of “back and forth through the focal points in the [national] agencies” to revise APRF operations [#03]. Similarly, the APRF successfully considered “ways of facilitating additional countries to participate in the Fourth Regional Ministerial Forum” ([63], p. 2), so that, beyond the original APRF members, there were an additional 20 countries represented in Manila in 2016 [64], thus occasioning the forum’s name change [65]. Both organisations also report positively on outputs relating more directly to climate and health. For example, in 2016 the APRF reported that 12, of its then 14 member states, had produced a NEHAP, with nine of the 12 including climate change as an environmental health issue [66]. Similarly, in 2017, the ADB’s Independent Evaluation Department reported on ADB projects making improvements to climate-related health determinants such as food security and safe water supply [67]. Both organisations also received positive ratings for engaging with stakeholders in relation to CCA and health. For example, the APRF has organised presentations on climate change and health for relevant ministers and senior government officials from 34 Asia-Pacific nations [65], and the ADB ran stakeholder consultation workshops regarding the direction of its SRCC project [55].

The results reported above relate to organisational goals, rather than to the specific initiatives that have been assessed here. A difference between the objectives of the initiatives, and organisational CCA and health outputs is that both initiatives include a focus on capacity building [55, 57], whereas the reported CCA and health outputs focus on readily measurable indicators such as access to safe water and sanitation or the production of policy documents [66, 67]. Building capacity is a targeted long-term outcome, in contrast to immediately measurable objectives, leaving a gap between initiative goals and organisational reporting.

## Discussion

Divided into four sections, the discussion triangulates the FAROCCCA results with academic literature and additional interview data, and argues that both the ADB’s and APRF’s CCA and health effectiveness could be improved if they worked together. First is an analysis of areas where each organisation’s work could be improved, and second is a rationale for a collaborative approach including an outline of how it could be achieved. Third is an analysis of potential barriers to an ADB and APRF collaboration, and a final section analysing FAROCCA’s performance and suggesting options for future research.

### Improving regional organisation effectiveness supporting CCA and health

Through the APRF, with support from WHO and UNEP, governments across the region can meet to discuss climate change and health issues, reflecting “their national interests and foreign policy priorities” ([68], p. 3). While this may appear positive, and similar to commentary about WHO more broadly (e.g. [6]), its potential is not being realised. While the lack of CCTWG meetings is indicative of low prioritisation of climate change and health, the re- endorsement of the CCTWG at the Ministerial Meeting of the APRF in October 2016 (researcher’s observation) suggests it is other factors that have inhibited activity. As described in the results, project management for the CCTWG could be improved through inclusion of specific and measurable targets as well as a monitoring and evaluation framework. Without these, incentives are lacking for government officials to invest in CCTWG discussions. The lack of incentives weakens linkages between the key personnel across the region, thereby undermining the peer-pressure based incentive structure used by the APRF.

Similarly, Biermann et al. ([69], p. 52) argued that “precisely state[d] goals, criteria and benchmarks for assessing progress” make international treaties more effective, and Dahle ([70], p. 40) found that a “lack of clear goals and tactics” can undermine initiatives. The APRF’s member governments and secretariat have recognised the benefits of an action-oriented approach [#06, #17]. Thus, the APRF has been encouraging its members to develop EHCPs and NEHAPs [66], but this approach has not yet been applied in the context of the CCTWG. Lack of targets and review framework make measuring progress challenging, thus constraining opportunities for process or system adjustments to reflect poor progress or changes in the external environment. These are areas of weakness that the APRF should address.

While the APRF’s CCTWG has struggled, the ADB has designed its own climate change and health project. The ADB designing the project was problematic because Southeast Asian government officials tend to view project design as a key aspect of achieving climate change-related outcomes [#20]. Additionally, perceived legitimacy is very important in Southeast Asia, where non-interference in individual countries internal affairs is a recognised ideal [11, 12]. In the ADB’s SRCC example, limited involvement of key government agencies in initial project design appears to have reduced its legitimacy in the eyes of key stakeholders, leading to discord and distrust and undermining the SRCC focus area of capacity building. From a resilience thinking perspective it makes sense to make changes in order to accommodate the needs of the RO’s clients (where it doesn’t undermine the ROs core identity and operating principles). Additionally, linked to multi-lateral development banks, such as the ADB, not having in-house technical capacity, and not being “well equipped to deliver at the local level” [#01], a consultancy firm was employed to manage the SRCC project, adding in additional levels of hierarchy and further reducing flexibility [71]. The ADB’s perceived legitimacy, which can impact on ability to achieve project outputs and outcomes, was negatively affected both by the use of an outside agency to implement the project, and by the need to control project design.

Another issue affecting the operational landscape is convergence of the mandates of the two organisations [10, Article 3, 13, p. 14, 50, Article 36(2)]. For ROs with membership and mandate overlaps, either coordination and cooperation, or duplication and competition for resources can result [68]. For the ADB and APRF a conscious coordination effort is important, because without it, future resource competition is likely for a combination of three reasons. Firstly, the ADB’s adaptation funding is predominantly externally grant-based [#12], as is the case for the SRCC project [62], so if the APRF seeks CCA and health funding it will bring the two organisations into competition, in line with Nolte’s [68] argument that a key to coordinated regional governance is mandate differentiation. Secondly, as part of their results-oriented approach the APRF has discussed seeking donor funding [#02], and thirdly, there is a strong possibility that any funds sought would be for climate change and health because of the increasing global prioritisation of CCA and health [8]. Despite the importance of collaborating, and the nine years since their mandates converged, cooperation to date has relied on individual initiative. For example, [#14] reported that brokering a relationship between the ADB and APRF is a constant problem, and that one individual had been ensuring continued communication between the two but is now constrained from doing that. From a resilience thinking perspective, this highlights a lack of institutional responsiveness to changing circumstances, and institutionalising and codifying ADB and APRF coordination would be a way to manage the risk of future inter-organisational resource competition. Resilience thinking also confirms that lack of certainty about whether the APRF will seek funding is not a reason for inaction in this area.

Effective coordination between ROs with mandate and membership overlaps means cooperating collaboratively in pursuit of the same goals, reflecting the importance that modern organisation theory places on communication processes. Because it is more than information sharing, this type of coordination requires incentives and frameworks [72], such as institutions to coordinate decentralised governments’ efforts [73]. Developing incentives, frameworks and institutions takes time, money as well as other resources. For this reason, there is a risk that investing in coordination may reduce an organisation’s effectiveness in the short-term more than its effectiveness is enhanced in the long-term. Despite the costs and potential risks, coordination benefits include reducing duplication of effort, such as the seaports built every 40–50 km along Vietnam’s central coast because of poorly coordinated provincial planning [74]. Investing in a collaborative and cooperative endeavour with another organisation needs to be well planned and formulated to ensure the costs are reasonable and able to be recouped.

Similar to the Vietnamese example above, poor ADB and APRF coordination of CCA and health support means duplicated effort in national agencies responsible for health and climate change concerns. For example, national agencies, such as the Vietnamese Health and Environment Management Agency and Cambodia’s Preventive Medicine Department, that work on APRF initiatives, are also working on the ADB’s SRCC project. If the ADB and APRF collaborated, and provided a single interface, it would significantly reduce the time and human resource investment for these national agencies to participate in, and administer, the projects. While collaboration would require an ADB and APRF investment, it would not violate either organisation’s charter or otherwise undermine their organisational identities, but would provide benefits for national environment and health focal agencies.

### Choosing to collaborate

Stemming from their complementarity, there are five reasons for the ADB and APRF to collaborate on CCA and health, despite incentivisation and framework costs. First, it would be drawing on experiences from other parts of the world. For example, to resolve mandate overlap issues, including duplication of effort, European ROs have focussed on developing common approaches in their work [75]. Second, as per the results in section “Initiatives 7.2” the ADB has project management expertise that the APRF lacks. In contrast, the APRF brings a visionary approach and methodology to building national ownership of health and environment concerns. Third, there are existing inter-organisational links. For example, members of national governments across Southeast Asia are involved in initiatives originating from both organisations, ADB personnel attended the APRF Ministerial Meeting in Manila in October 2016 [64], ADB consultants have attended APRF Health Impact Assessment TWG meetings [#14], and the ADB provided financing when the APRF was established in 2007 [#02]. Fourth, in line with Scott’s “mutual dependency” [35] and Brosig’s rational choice [76], the ADB strength in financial resource acquisition could be partnered with the APRF’s high levels of perceived legitimacy and long-term expertise in public health and climate change from across the region [#14]. Finally, a CCA and health collaboration would formalise the convergence of the two CCA and health mandates. A summary of the reasons for the two organisations to collaborate is included in Table 2, below:

**Table 2:** Reasons for an ADB/APRF collaboration

	Reasons	ADB (SRCC) Details	APRF (CCTWG) Details
1	Complementary Strength	ADB brings project management strengths	APRF brings vision and an ownership-building methodology
2	Complementary Strength	ADB has access to significant direct and grant-based finances	APRF has higher levels of perceived legitimacy, and brings ‘in-house’ expertise in public health and climate change
3	Historical and contemporary links	Existing inter-organisation links, such as the same national level personnel involved in both initiatives as well as ADB personnel attending APRF events	
4	Risk management	A collaborative partnership would formalise a convergence between the mandates of the two organisations, thus helping to manage the risk of future inter-organisational competition for funding	

To ensure success, a detailed work plan defining organisational roles and responsibilities and the goals of the cooperative effort will need to be developed. To satisfy the framework requirement of Resurreccion et al. [72] and Saito [73], each organisation should assign coordination responsibilities to one department, incentivised with reporting requirements that include assessment criteria. Building on the similarities between their climate change and health mandates, and thus simplify initial coordination complexities, the CCTWG should be the entry point for an ADB-APRF collaboration. As an initial collaborative step, the CCTWG and the ADB’s Sustainable Development and Climate Change Department could foster cross-membership, and work towards a standard administrative interface, such as established between the European Union and the Council of Europe [77]. There is a clear window of opportunity for this, as the CCTWG has not met since December 2013, and is in need of re-invigoration following its re-endorsement at the APRF’s 2016 Ministerial Meeting. Collaboration will allow the ADB to trial an alternative model of supporting national CCA and health measures, collaborating with national governments both to define the terms of their relationship and to set priorities, thereby enhancing the ADB’s perceived CCA and health input-legitimacy (e.g. [24, 25]). With the CCTWG as an entry point, the organisations can each contribute in their area of strength, and early collaboration success re-invigorating the APRF’s CCTWG would support further collaboration on other Southeast Asian environment and health concerns.

### Constraining factors

There are a number of hurdles requiring negotiation to make a CCA and health collaboration viable. First are the existing coordination difficulties between the ADB and APRF [#14]. Institutionalisation of coordination requirements is more durable than relying on individual initiative, and will avoid coordination gaps when no-one takes personal initiative. Coordination reporting requirements will help ingrain coordination habits, thus helping to gradually overcome resistance. Second, the ADB has a lack of flexibility in how it deals with national government agencies [#13, #14]. However mandate convergence shows that ADB objectives can shift, and the ADB does work with outsiders, thus with suitable arguments institutional support is likely to be forthcoming. Third, the APRF visionary approach only includes health and environment ministries. This is problematic because, for example, in Vietnam the planning ministry decides on national funding priorities [#22]. Therefore the APRF’s ability to support the development of national environment and health ownership is constrained because key decision-making ministries/bodies are not involved, and may not have a good awareness of environment and health concerns. For example, Resurreccion et al. [72] argued that Vietnam’s planning and investment ministry does not have climate change expertise, and Myanmar’s energy ministry mandate is energy security, with climate change just a minor consideration [#21], leaving little space for CCA and health concerns. Fourth, relatedly, the APRF faces funding constraints, with available funds “mostly used for the travel of participants from developing countries to come to these meetings” [#02], leaving little room for increasing the number of ministries involved. Collaborating with the ADB could help relieve some of the APRF’s budgeting limitations allowing, for example, involvement of other key ministries.

### FAROCCA performance and future research

This research used a modified form of FAROCCCA, which was originally developed to assess ROs supporting CCA in small island developing states. Its use in this research shows it can be adjusted across geographies and is not discipline-specific. There were some limitations in the use of the modified FAROCCCA. The researcher being an outsider minimised the likelihood of researcher bias, however the lack of depth of relationships with interviewees meant that the perceptual information gathered was limited. Several of the modified FAROCCCA’s sub-components included perceptual indicators, but the structure, systems and processes sub-component was most affected, with seven of eight indicators being perceptual. In order to assess these without loss of assessment objectivity the modified FAROCCCA could be used by an officially sanctioned independent evaluator. Other than this observation, use of the modified FAROCCCA provided significant insights into the APRF’s and ADB’s CCA and health endeavours. While many of the results were expected, others, such as the ADB’s monitoring and evaluation results, were surprising. The legitimacy results suggest that FAROCCCA could be further modified to include more nuanced legitimacy indicators.

This research into the role of ROs supporting adaptation and health across Southeast Asia should stimulate and inform debate about the most effective ways for regional engagement with CCA and health issues. Four options for future research are identified here:

- As many Southeast Asian countries are confronting decentralisation issues [78–80], future research could investigate the possibility that the CCTWG could support the strengthening of decentralisation programs, though its focus on CCA and health.
- Organisation sanctioned re-application of the modified FAROCCCA including the use of the perceptual indicators would provide additional information on the organisations’ input legitimacy.
- This research highlights regional organisational legitimacy implications as an area for further investigation.
- Some individuals naturally seek to coordinate and work with others, but it is not a universal trait. Future research into coordination should include not just suitable mechanisms, but also how to identify and attract individuals who will enhance the outputs and outcomes of those coordination mechanisms.

## Conclusion

This research makes four primary contributions to the academic literature on CCA and health. First, by using a modified framework to qualitatively assess regional contributions of two organisations to adaptation and health highlighted FAROCCCA’s broad applicability across geographies and sectors. Second, this qualitative assessment of two regional models shows that there are existing weaknesses in both project-based and governance-based models of regional support to the national level in Southeast Asia, which if addressed have the potential to improve adaptation and health outcomes across the region. Third, this research demonstrated that these weaknesses could be addressed through institutionalised coordination and collaboration. Doing so could address perceived legitimacy issues that were highlighted as an issue in this research, with these issues being of high importance, particularly because non-interference in individual nations affairs has been codified as a regional Southeast Asian ideal. Fourth, this research demonstrated that, for the ADB and the APRF, institutionalising coordination between the ADB’s Sustainable Development and Climate Change Department and the APRF’s CCTWG would be a good starting point. The research presented in this paper discusses coordination problems, which transcend regions and governance arrangements, and is thus should be of interest to regional organisations working in other areas of the world as well as in sectors other than adaptation and health.

## Additional files

Additional file 1: Original and modified FAROCCCA indicators with qualitative CCA and health results for the APRF and ADB. (DOCX 250 kb)

Additional file 2: Modified FAROCCCA applied to the Asia-Pacific Regional Forum on Health and Environment (APRF). (DOCX 89 kb)

Additional file 3: Modified FAROCCCA applied to the Asian Development Bank. (DOCX 51 kb)
