# Supplementary material for: Regional organisations supporting health sector responses to climate change in Southeast Asia
Source: Global Health. 2018 Aug 3;14:80. doi: 10.1186/s12992-018-0388-z (PMC6091073; doi:10.1186/s12992-018-0388-z)
Supplement: Supplementary file 1 — Original and modified FAROCCCA indicators with qualitative CCA and health results for the APRF and ADB. (DOCX 250 kb) [file 12992_2018_388_MOESM1_ESM.docx]

**Additional file 1: Original and modified FAROCCCA indicators with qualitative CCA and health results for the APRF and ADB**

| **RATING SYSTEM** | |
| --- | --- |
| 🗷 | No |
| 🞚 | To some extent |
| 🗹 | Yes |
| (NE) | No evidence |
| (PI) | Perceptual indicator or indicator not rated in this paper |

| **SUB-COMPONENT** | **CCA INDICATORs**  **(As per (Robinson and Gilfillan 2017)** | **CCA and HEALTH INDICATORS**  **(FOR SOUTHEAST ASIA)** | **APRF** | **ADB** |
| --- | --- | --- | --- | --- |
| ***COMPONENT 1. Input Effectiveness*** | | | | |
| 1. Goals | 1. Climate change adaptation was an initial goal of the organisation. | 1. Health sector adaptation to climate change was an initial goal of the organisation/forum./project | 🗹 | 🗷 |
|  | 1. Climate change adaptation is a current goal of the organisation. | 1. Health sector adaptation to climate change is a current goal of the organisation/forum/project. | 🗹 | 🗹 |
|  | 1. The current strategic plan contains specific climate change adaptation objectives. | 1. Current strategies/plans contain specific climate change adaptation objectives for the health sector. | 🗹 | 🗷 |
|  | 1. There is no other regional organisation with similar climate change adaptation goals. | 1. There is no other regional organisation/forum/project with similar climate change adaptation goals for the health sector. | 🗷 | 🗷 |
| 1. Governance and leadership | 1. The Board provides visionary leadership and strategic direction. | 1. There is visionary leadership and strategic direction for the organisation/forum/project. | 🗹 | 🗷 |
|  | 1. The organisation evaluates organisational performance at least annually. | 1. There is an evaluation of organisational/forum/project performance at least annually conducted by the organisation/forum/project. | 🞚 | 🗹 |
|  | 1. Executive management (can also include members of the Board/Governing Body) decision-making is done by consensus or majority vote. | 1. Organisational/forum/project decision-making is done by consensus or majority vote. | 🗹 | 🗹 |
|  | 1. Executive management staff (can also include members of the Board/Governing Body) are qualified and/or equipped to achieve the goals of the organisation. | 1. Organisational/forum/project personnel are qualified and/or equipped to achieve the goals of the organisation/forum/project. | 🗹 | 🗹 |
|  | 1. Executive management staff disclose potential conflicts of interest. | 1. Organisation/forum/project personnel are required to disclose potential conflicts of interest. | (PI) | (PI) |
|  | 1. The organisation attracts, retains and develops talent. | 1. The organisation/forum/project has mechanisms to attract, retain and develop talent. | 🞚 | 🗹 |
|  | 1. Leaders create a dynamic organisational culture, making the organisation a desirable place to work. | 1. Leaders create a dynamic organisational/forum/project culture, making it a desirable place to work. | (PI) | (PI) |
|  |  | 1. The organisation/forum/project is perceived by stakeholders as legitimate. | 🗹 | 🞚 |
| 1. Resources | 1. There are staff members exclusively dedicated to climate change adaptation. | 1. There are organisational/forum/project personnel exclusively dedicated to health sector adaptation to climate change. | 🗷 | 🞚 |
|  | 1. Staff are qualified and have experience in climate change adaptation. | 1. Personnel are qualified and have experience in health sector adaptation to climate change. | 🗹 | 🗹 |
|  | 1. Staff are qualified and have experience in project/program management. | 1. Personnel are qualified and have experience in project/program management. | 🗹 | 🗹 |
|  | 1. Staff participate in ongoing training programs. | 1. Personnel participate in ongoing training programs. | 🗹 | 🗹 |
|  | 1. Staff performance is appraised (formally or informally) at least annually. | 1. Personnel performance is appraised (formally or informally) at least annually. | (NE) | 🗹 |
|  | 1. The organisation has untied funding. | 1. The organisation/forum/project has untied funding. | 🞚 | 🗹 |
|  | 1. There is evidence that the organisation/forum/project includes a component exclusively focussed on health sector adaptation to climate change. | 1. There is evidence that the organisation/forum/project includes a component exclusively focussed on health sector adaptation to climate change. | 🗹 | 🗹 |
|  | 1. External funding to the organisation has increased over the past 5 years. | 1. External funding to the organisation/forum/project has increased over the past 5 years. | 🗷 | 🗷 |
|  | 1. The organisation has multiple funding sources. | 1. The organisation/forum/project has multiple funding sources. | 🞚 | 🗹 |
|  | 1. The organisation has financial reserves. | 1. The organisation/forum/project has financial reserves. | 🗷 | 🗹 |
|  | 1. The organisation has sufficient technological resources (e.g. intellectual property rights, patents, copyright, software licences etc.) to carry out its climate change adaptation mandate. | 1. The organisation/forum/project has sufficient technological resources (e.g. intellectual property rights, patents, copyright, software licences etc.) to carry out its climate change adaptation and health mandate. | (PI) | (PI) |
| 1. Structure, systems and processes | 1. There is a low degree of hierarchy (i.e. few hierarchical levels). | 1. The organisation/forum/project has a low degree of hierarchy (i.e. few hierarchical levels). | (PI) | (PI) |
|  | 1. The organisation has a human resource management system that supports the shaping of organisational culture and staff recruitment, training, development and retention. | 1. The organisation/forum/project has a human resource management system that supports the shaping of organisational culture and staff recruitment, training, development and retention. | (PI) | (PI) |
|  | 1. There is a financial management system that meets International Financial Reporting Standards (IFRS) or its equivalent. | 1. The organisation/forum/project has a financial management system that is internationally recognised. | 🞚 | 🗹 |
|  | 1. The organisation applies risk management principles in its decision-making processes. | 1. The organisation/forum/project applies risk management principles in its decision-making processes. | (PI) | (PI) |
|  | 1. The organisation has a centralised, user-friendly internal data management system. | 1. The organisation/forum/project has a centralised, user-friendly internal data management system. | (PI) | (PI) |
|  | 1. The organisation has a user-friendly project/program management system (e.g. that supports staff to identify, schedule and track resources etc.). | 1. The organisation/forum/project has a user-friendly project/program management system (e.g. that supports personnel to identify, schedule and track resources etc.). | (PI) | (PI) |
|  | 1. There are mechanisms that support vertical and horizontal communication. | 1. There are mechanisms that support both vertical and horizontal communication within the organisation/forum/project | (PI) | (PI) |
|  | 1. There are internal dispute resolution protocols. | 1. The organisation/forum/project has internal dispute resolution protocols. | (PI) | (PI) |
| 1. Research and collaboration capacity | 1. The organisation has plans and policies that support research. | 1. The organisation/forum/project has plans and policies that support research. | 🗹 | 🗹 |
|  | 1. There are organisational funds allocated for research. | 1. There organisation/forum/project has funds allocated for research, or facilitates access to research funds. | 🗹 | 🗹 |
|  | 1. The organisation has equipment, expertise and/or resources (e.g. access to journal articles etc.) for research. | 1. The organisation/forum/project has equipment, expertise and/or resources (e.g. access to journal articles etc.) for research, or is able to facilitate access to research related resources | 🗹 | 🗹 |
|  | 1. The organisation’s current strategic plan (or a similar document) outlines plans for collaboration with multiple stakeholders on adaptation-related initiatives. | 1. The current organisational/forum/project strategic plan (or a similar document) outlines plans for collaboration with multiple stakeholders on health sector adaptation-related initiatives. | 🗹 | 🗹 |
| ***COMPONENT TWO: Project/Initiative Effectiveness*** | | | | |
| 1. Needs and goals | 1. The project documents contain evidence that the project/program fills an existing need with relation to climate change adaptation. | 1. There is evidence that the project/program/forum initiative is filling an existing need with relation to climate change adaptation and health. | 🗹 | 🗹 |
|  | 1. The project/program’s adaptation components could be considered ‘transformational’ (i.e. the project/program focusses on “larger, more profound system changes” and requires a “paradigm shift” in the way it is framed and implemented). | 1. The adaptation focus of the project/program/forum’s initiative could be considered ‘transformational’ (i.e. there are marked shifts in the way the health sector is framed and the way it operates, leading to “larger, more profound system changes”.). | 🞚 | 🞚 |
|  | 1. Climate change adaptation is a goal of the project/program. | 1. Climate change adaptation and health is a goal of the project/program/forum initiative/activity | 🗹 | 🗹 |
|  | 1. The project/program’s goals reflect the long-range impacts of climate change. | 1. The initiative/activity goals reflect the long-range impacts of climate change. | 🗹 | 🗹 |
|  | 1. The project/program’s objectives relating to climate change adaptation are specific, measurable, achievable, realistic and time-bound (SMART). | 1. The initiative/activity’s objectives relating to climate change adaptation and health are specific, measurable, achievable, realistic and time-bound (SMART). | 🗷 | 🗹 |
|  | 1. Member Countries were involved in developing the climate change adaptation components of the project/program. | 1. Member Countries were involved in developing the climate change adaptation and health components of the initiative/activity | 🗹 | 🗹 |
| 1. Scope | 1. The project/program addresses multiple climate or climate-induced vulnerabilities (e.g. vulnerability to sea-level rise, increased sea surface and air temperature, changing rainfall patterns etc.). | 1. The initiative/activity addresses multiple climate or climate-induced and health-related vulnerabilities (e.g. vulnerability to sea-level rise, increased sea surface and air temperature, changing rainfall patterns etc.). | 🗹 | 🗹 |
|  | 1. The project/program addresses multiple non-climate-induced vulnerabilities (e.g. poverty, deforestation etc.). | 1. The initiative/activity addresses multiple non-climate-induced health-related vulnerabilities (e.g. poverty, deforestation etc.). | 🗹 | 🗹 |
| 1. Logic, design and adequacy | 1. The logic/design of the project/program’s climate change adaptation components is evidence-based, in the context of SIDS. | 1. The logic/design of the initiative/activity’s climate change adaptation and health components is evidence-based and contextualised. | 🗹 | 🗹 |
|  | 1. The project documents contain evidence that the logic/design of the project/program’s climate change adaptation components is an effective means to achieve its objectives. | 1. There is evidence that the logic/design of the initiative/activity’s climate change adaptation and health components is an effective means to achieve its objectives. | 🗷 | 🗹 |
| 1. Resources | 1. Staff members are assigned exclusively to the project/program. | 1. Personnel are assigned exclusively to the initiative/activity. | 🗷 | 🞚 |
|  | 1. The project/program team includes staff members with qualifications and experience in climate change adaptation. | 1. Personnel involved with implementing the initiative/activity’s adaptation components have qualifications and experience in climate change adaptation. | 🗹 | 🗹 |
|  | 1. The project/program team includes staff members with qualifications and experience in project/program management. | 1. Personnel involved with implementing the initiative/activity’s adaptation components have qualifications and experience in project/program management. | (NE) | 🗹 |
|  | 1. The project documents contain evidence that there are sufficient staff members to achieve the project/program objectives. | 1. There is evidence that there are sufficient personnel to achieve the objectives of the initiative/activity. | (NE) | 🗹 |
|  | 1. The project documents contain evidence that there is sufficient funding for the project/program’s climate change adaptation components. | 1. There is evidence of sufficient funding for the initiative/activity’s climate change adaptation components. | 🗷 | 🗹 |
| 1. Technical efficiency | 1. The project documents contain evidence that the project/program provides value for money (cost vs. outputs). | 1. There is evidence that the initiative/activity provides value for money (cost vs. outputs). | 🗹 | 🞚 |
| 1. Implementation | 1. The project/program’s climate change adaptation components are implemented, as proposed. | 1. There is evidence that the initiative/activity’s climate change adaptation and health related components have been implemented as proposed. | 🞚 | 🗷 |
| 1. Monitoring and evaluation | 1. The project/program is internally monitored and evaluated. | 1. There is evidence that the initiative/activity is internally monitored and evaluated. | 🗷 | 🗷 |
|  | 1. The project/program is externally monitored and evaluated. | 1. There is evidence that the initiative/activity is externally monitored and evaluated. | 🗷 | 🞚 |
| 1. Sustainability | 1. There are sustained outputs from the project/program. | 1. There is evidence of sustained outputs from the initiative/activity. | (NE) | (NE) |
| ***COMPONENT THREE: Output Effectiveness*** | | | | |
| 1. Goal attainment | 1. There is evidence in the most recent annual report or evaluation that the climate change adaptation-related objectives of the organisation are being achieved. | 1. There is evidence in the most recent annual report or evaluation that the climate change adaptation and health-related objectives of the organisation/project/forum are being achieved. | 🗹 | 🗹 |
| 1. Research and knowledge management | 1. The organisation produces and/or publishes research that is relevant to climate change adaptation at least annually. | 1. The organisation/project/forum produces and/or publishes research that is relevant to climate change adaptation and health at least annually. | 🗹 | 🗹 |
|  | 1. The organisation makes climate change adaptation-relevant research publicly available. | 1. The organisation/project/forum makes climate change adaptation and health related research publicly available. | 🗹 | 🗹 |
| 1. Collaboration and advocacy | 1. There is evidence that the organisation collaborates with multiple stakeholders to undertake climate change adaptation-related activities. | 1. There is evidence that the organisation/project/forum collaborates with multiple stakeholders to undertake climate change adaptation and health-related activities. | 🗹 | 🗹 |
|  | 1. The organisation advocates for political, financial and/or other climate change support for its Member Countries in various fora at different scales. | 1. The organisation/project/forum advocates for political, financial and/or other climate change and health related support for its Member Countries in various fora at different scales. | 🗹 | 🗹 |
| 1. Education and training | 1. The organisation undertakes climate change adaptation stakeholder and/or public awareness activities. | 1. The organisation/project/forum undertakes climate change adaptation and health related stakeholder and/or public awareness activities. | 🗹 | 🗹 |
|  | 1. The organisation develops and/or implements training programs for stakeholders in issues related to climate change adaptation. | 1. The organisation/project/forum develops and/or facilitates the implementation of training programs for stakeholders in issues related to climate change adaptation and health. | 🗹 | 🗹 |
| 1. Specialised advisory services | 1. The organisation provides specialised climate change adaptation-related advice to Member Countries and/or other stakeholders. | 1. The organisation/project/forum provides specialised climate change adaptation and health related advice to Member Countries and/or other stakeholders. | 🗹 | 🗹 |

Robinson, S.-a. and D. Gilfillan, 2017. Regional organisations and climate change adaptation in small island developing states, *Regional Environmental Change,* **17**: 989-1004. Available at: <http://dx.doi.org/10.1007/s10113-016-0991-6> (accessed June 20, 2016).
