# Supplementary material for: Regional organisations supporting health sector responses to climate change in Southeast Asia
Source: Global Health. 2018 Aug 3;14:80. doi: 10.1186/s12992-018-0388-z (PMC6091073; doi:10.1186/s12992-018-0388-z)
Supplement: Supplementary file 2 — Modified FAROCCCA applied to the Asia-Pacific Regional Forum on Health and Environment (APRF). (DOCX 89 kb) [file 12992_2018_388_MOESM2_ESM.docx]

**Additional file 2: Modified FAROCCCA applied to the Asia-Pacific Regional Forum on Health and Environment (APRF)**

| **RATING SYSTEM** | |
| --- | --- |
| 🗷 | No |
| 🞚 | To some extent |
| 🗹 | Yes |
| (NE) | No evidence |
| (PI) | Perceptual indicator or indicator not rated in this paper |

| **SUB-COMPONENT** | **INDICATOR** | **EVIDENCE** | **RATING** |
| --- | --- | --- | --- |
| ***COMPONENT 1. Input Effectiveness*** | | | |
| 1. Goals | Health sector adaptation to climate change was an initial goal of the organisation/forum./project | “Governments should address the health impacts and implications of the following priority areas of environmental concern at the local, national, regional and global levels:  • Air quality  • Water supply, hygiene and sanitation  • Solid and hazardous waste  • Toxic chemicals and hazardous substances  • Climate change, ozone depletion and ecosystem changes  • Contingency planning, preparedness and response in environmental health emergencies” (RFEH, 2007a, Article 3)(This is the RFEH Charter)  ***The APRF charter clearly addresses the concern of health impacts relating to a changing climate*** | 🗹 |
|  | Health sector adaptation to climate change is a current goal of the organisation/forum/project. | “Understanding that the environment in which we live has both pathogenic and salutary effects on our health and that our common goal is the attainment of healthy lives through healthy environments and sustainable development;  Recognizing the need to achieve that goal by ensuring clean air, safe and adequate water, environmentally sound treatment and safe disposal of waste, safe management of chemical substances, adequate response and adaptation to climate change, efficient and effective actions in environmental health emergencies, and assessment of health impacts of these and other environmental hazards;” (RFEH, 2013c, p. 1).  “We are bringing the policy-makers – we are presenting the inter-linkages between health and environmental issues, which includes the different types of pollution, and that is impacting health, including the climate change issues as well, when we are talking about the carbon pollution, which is increasing the temperature, as well bringing the erratic behavior of rainfall, impacting the production of food and then how those are linked with the health, including the nutrition aspect of the population” [#19].  ***The Kuala Lumpur declaration on climate change re-iterates that adapting to climate change is a key component of environmental health concern. This is supported by [#19]’s comment.*** | 🗹 |
|  | Current strategies/plans contain specific climate change adaptation objectives for the health sector. | 2014-2016 workplan includes a climate change and health activity, with timelines and outputs, however outputs are vague and don’t show strong links to the goal – eg. Activity is to “To strengthen national and regional programmes on climate change impacts on health”, and output 2 is “Side event at the 2014 Asia Pacific  Adaptation forum in KL” (Regional Forum on Environment and Health in Southeast and East Asian Countries, 2014, p. 13)  ***There is a specific CCA objective (strengthening national and regional programs)*** | 🗹 |
|  | There is no other regional organisation/forum/project with similar climate change adaptation goals for the health sector. | GEF funded project - Building Resilience of Health Systems in Asian LCDs to Climate Change, in Bangladesh, Cambodia, Lao PDR, Myanmar, Nepal, Timor-Leste, implemented by UNDP with support from WHO (GEF, 2016).  [Note: Despite this project, GEF’s establishment document doesn’t include the word health (GEF, 2015b), and GEF’s strategy to 2020 only mentions human health in relation to chemical pollution (GEF, 2015a).]  “The Asian Development Bank (ADB) has approved a pioneering regional technical assistance initiative, with finance from the Nordic Development Fund (NDF), to help Cambodia, the Lao People’s Democratic Republic (Lao PDR), and Viet Nam respond to climate-induced health threats” (ADB, 2015, online).  And an overall goal for the ADB:  According to the ADB’s ‘Strategy 2020’, “ADB will also help DMCs [developing member countries] adapt to the unavoidable impacts of climate change—including those related to health” (ADB, 2008).  ***The ADB’s strategy 2020 includes similar health and adaptation goals***  ***The GEF does not have a direct mandate for adaptation and health in its strategic documents, but is funding at least one project with a similar goal in the region.*** | 🗷 |
| 1. Governance and leadership | There is visionary leadership and strategic direction for the organisation/forum/project. | The leadership of the secretariat has been visionary. There has been a focus on generating ownership among member states for the goals of the regional forum through the use of peer pressure – member states knowing a meeting is coming up and that their peers will have (for example) produced an Environmental Health Country Profile (EHCP):  “rather than being seen as we are pushing, is to find mechanism where they feel that they [the member states – particularly developing countries] have to do, so for example, like we inviting them for a regional meeting, to update – is a kind of informal subtle way for them to do the country profile. So, we – I don’t know, we plan to call for some kind of meeting in WHO, so that when they go, Oh, Nasir, ah Hassan is calling for country profile or data sheet” [#06]  To qualify this, not everyone involved feels this way. For example, in an informal conversation, I was told that getting the ministerial meeting to sign off on the environmental health declaration was only useful because it would then give WHO or UNEP additional leverage to get national approval for running environmental health projects within participating countries.  ***Looking for ways to encourage and support national government personnel to take ownership of environmental health issues is visionary for two reasons:***   1. ***National ownership will lead to better long-term success than ideas being pushed from the outside.*** 2. ***Institutionalising environmental health in national governments will help break down sectoral silos.*** | 🗹 |
|  | There is an evaluation of organisational/forum/project performance at least annually conducted by the organisation/forum/project. | “Operations: the high-level official meeting will review the midterm progress of the Regional Forum during the past 18 months based on the current work plan” (RFEH, n.d., Article 12)  “Role of Secretariat: the secretariat is responsible to prepare the work plan midterm progress review, interim priorities and targets of the Regional Forum for approval of the high-level officials” (RFEH, n.d., Article 16).  “As you heard, our plan is to spend at least the first half of 2017 to really evaluate what regional forum so far has achieved, and to come up with more detailed plans of the regional implementation. And even revisit some of the mechanisms of the regional forum, like thematic working groups, so we have something that will continue to serve most efficiently the regional forum. I think it would be very intense six months next year, to revise the implementation plan, revisit the whole different mechanism under the regional forum, but after that we will be doing once a year at least – the joint evaluation between WHO – UNEP and that will be part of secretariat service we provide to high level officials and the regional forum itself.” [#17]  ***There is evidence of the intention to conduct regular evaluations, however these evaluations are not present on the forum website. The most recent evaluation available on the website is for the period 2013-2016 (a three year period).*** | 🞚 |
|  | Organisational/forum/project decision-making is done by consensus or majority vote. | “Recommendations and decisions of the scientific panel shall be decided by consensus where possible, with all core members supporting a particular point of view. Where consensus is not reached, all recommendations shall be presented to the Regional Forum and the lack of consensus noted” (RFEH, n.d.).  The charter for the Forum implies that decisions are made by consensus:  “The Forum shall: […]  (c) formulate recommendations on the implementation of the consensus established by the Forum;” (RFEH, 2007a).  ***Researcher observations of the workings of the forum indicate decision-making by consensus, and this is backed up by the implication in the forum charter, as well as the guidance for the scientific panel on how its decisions should be made.*** | 🗹 |
|  | Organisational/forum/project personnel are qualified and/or equipped to achieve the goals of the organisation/forum/project. | “The meetings will be attended by the Ministers of Health and Environment and high-level government officials from the Ministries in charge of environment and health issues in the 14 countries, representatives from relevant UN agencies, Asian Development Bank, World Bank and other international partners, government officials of other Asian countries and members from academia, professional associations and non-governmental organisations” (RFEH, 2007c, Participants).  “Each of the 14 member countries designates a national communication focal point (NFP), one from the Ministry of Health and one from the Ministry of Environment” (RFEH, 2007b, Article 4).  “The Ministers of Environment and the Ministers of Health of member countries will meet at a minimum of once every three years to:  a) provide overall guidance and strategic direction consistent with the vision, goal and objectives of the Regional Forum;  b) determine priority areas and associated actions to improve health and the environment in the region;  c) enhance coordination and cooperation among countries and partner agencies in addressing identified environment and health priorities;  d) review and discuss the reports, scientific evidence and proposed recommendations of the Secretariat;  e) consider recommendations of member countries regarding future work and strategic directions; and  f) invite and engage other countries or relevant entities to be members and participate in the work of the Regional Forum” (RFEH, 2013b, p. 6).  ***Ministers of Health and Environment are at the top tier of government, and as such have significant resources at their disposal: personnel in the ministry (Human Resources), financial (Ministry budgets), information (about national priorities and what their ministries can and cannot achieve). They are also likely to have good networks associated with their respective portfolios (e.g. with development partners, private companies they could partner with).***  Lesley Onyon is listed as a staff member taking leave, in the OECD’s Environment, Health and Safety News (Newsletter) in 2001 ***(OECD, 2001). Indicating over 15 years experience in environment and health.***  Nassir Hassan (Responsible Officer for the APRFHE (APRFHE, 2016a)) was listed as an expert resource (as an environmental engineer) in the Cambodia 2011 Human Development Report, which focussed on resilience and people’s well-being in the face of climate change (MoE Cambodia and UNDP Cambodia, 2011).  ***Both Dr Lesley Onyon and Dr Mhd. Nasir Hassan (listed as secretariat members) have experience and expertise in environmental health.*** | 🗹 |
|  | Organisation/forum/project personnel are required to disclose potential conflicts of interest. |  | (PI) |
|  | The organisation/forum/project has mechanisms to attract, retain and develop talent. | “the second one [Ministerial Forum] was carried out in Jeju […] [in 2010] and that was a time that we that we feel that the regional forum is not getting the momentum, one of the main reasons is that there is no resource, and ASEAN did not put official commitment. Resources is a big issue, […] financial resources, but it has also maybe a lack of technical impact. So in Jeju, Korea, the Ministers ask, establish a committee to review the regional forum, review include closing […], we found out from member states – all these fourteen countries, saying that not only is it important, but they want to pursue this regional forum. It a very important platform in spite of challenges and so on and so forth. And the third one we had in Kuala Lumpur in 2013 – the third one – everyone say we want this to go on, with probably an improved direction, probably more focus areas of work” [#06]  ***Cambodia and Vietnam both have weak health sectors, and this can impact on their ability to attract the best talent. Eg***. Some staff in the health ministry “are not happy because often there are many issues – they don’t have any people in charge – some document from up there came from minister of health and nobody looks at those documents because they don’t have the responsibility they don’t have the benefits” [VN#04].  “Yes, [the finance ministry] is a more attractive place to work [than the health ministry] because the staff they have more income” [VN#04]  ***Similarly, Cambodian respondents discussed the lack of funding for climate change and health activities, so that in the first year of a five year action plan on climate change there have been no climate change and health activities*** [CB#05,CB#06]  ***In Contrast, Myanmar has a strong health sector, although senior health ministry personnel have left the health ministry to take up senior roles in other sectors such as social welfare*** (identifying reference).  The secretariat for the Forum is “quite small – it just me, it was me on the UNEP side, and on the WHO side they have people, two people, one from their, each of their regional offices” and for me “it’s a part time role” and “until very recently I was working as the UNEP member of the joint WHO UNEP secretariat to the regional environment and health forum” [#02].  ***At one level the organisation isn’t effectively attracting and retaining staff. For example, weak health sectors in Cambodia and Vietnam, health staff leaving health ministry in Myanmar and secretariat staff leaving.***  ***At another level, the forum is clearly providing some value to its members, as the ministerial forum in Jeju (2010) indicated they wanted it to continue, and also from 14 countries at the ministerial forum in 2013 (Kuala Lumpur) increased to 34 countries represented in the 2016 ministerial forum (manila) – indicating a high interest in the region in environment and health related issues and how to address them.*** | 🞚 |
|  | Leaders create a dynamic organisational/forum/project culture, making it a desirable place to work. |  | (PI) |
|  | The organisation/forum/project is perceive by stakeholders as legitimate. | “there was this taskforce that was about revising the whole operation. So there was a questionnaire that was sent to all the member states focal points, all agency focal points, and we then compiled it with all the recommendations and sent it back. So there was a lot of back and forth through the focal points in the agencies […] once we got this back, as a secretariat it was compiled into a report that was then again submitted to the focal points and to the ministries for – with a few recommendations coming from the questionnaires on how to re-structure. And then this would be the main background document for discussion in the high level meeting. […] [O]ne of the things that happened in this process of revising the operation was to – replace the advisory report with a scientific panel. So that would give more hard evidence – let’s say substantial information for the high level officials to decide. And then whatever came out of the high level officials discussions would be fed into the ministerial meetings for final approval.” [#03]  ***The process outlined here is a clear indication of national focal agency involvement in developing a meeting agenda item on re-structuring the APRF operations. This participation from member states builds the APRF’s*** “right to be and do”, ***so that it is*** “lawful, admissible and justified in its chosen course of action” (Edwards in Collingwood, 2006).  ***Further evidence that stakeholders view the APRF as legitimate is provided by*** [#10]: “from the [last] high level meeting […] WHO and UNEP asked […] members to develop or update the environmental [health] country profile, and then send back […] – we already send”. | 🗹 |
| 1. Resources | There are organisational/forum/project personnel exclusively dedicated to health sector adaptation to climate change. | This is dependent on the country. For example, MM health ministry has a disaster response division. Disasters include earthquakes as well as other non-climate-related events, but also floods, cyclones and droughts which are increasing in severity and number in Myanmar (identifying reference).  “About the capacity of the ministry of health, maybe I would just comment that the department of preventative medicine, who you have gotten to know, there is probably seven eight staff there, headed by Dr [Prak] Piseth Raingsey, the director, her deputy is Dr Hero. And when you look at.. ..this is their baby [indicating the projects on the summary sheet] this is their baby, that's a couple of million dollars over the next few years, and even to some extent this [GIZ] is theirs, and this [other GIZ] as well, although some of it's more through CNM, which really should also be coordinated by preventative medicine department. They, not to say they won't grow to suit to the task, but you can't just put multi-million dollar flow into a department that's doing six or eight or ten things now, without some corresponding increase in capacity there” [C#07]  In Vietnam, all climate change related activities are the responsibility of MONRE, however climate change and health is the responsibility of the Vietnam Health and Environment Management Agency (VIHEMA), which has several divisions.  At UNEP: “The linnkages [with the regional forum’s TWG on climate change] are actually through me, [but do] we have a specific person to deal with this, the answer is no.” [#19]  The secretariat for the Forum is “quite small – it just me, it was me on the UNEP side, and on the WHO side they have people, two people, one from their, each of their regional offices” and for me “it’s a part time role” [#02]  “We have decided as regional office that this environment and health forum is very important work to focus [on] […] “Now that the global mandate has caught up, in coming years we have more motivation and the reason to further strengthen our [support] to the countries on environmental health. So, until now, my job is not to coordinate environmental health work, my job is the sub-regional coordinator on the chemicals and waster, but I add on my job description that I can dedicate up to 20-30% of my time on the issue of regional significance. And for this regional office they have decided that the majority of my time (of that 20-30%) should be allocated to environment and health coordination” [#17].  Nassir Hassan is the responsible officer for the APRFHE, as part of his role as “Coordinator Health and the Environment Division of NCD and Health through the Life-Course [at] WHO Regional Office for the Western Pacific” (APRFHE, 2016a: 38).  ***The forum secretariat does not have dedicated personnel***  ***While the information is not totally clear, it is unlikely that developing member countries have dedicated personnel given their resource constraints (e.g. [C#07]’s quote about seven or eight staff, with already eight or ten responsibilities) and the number of development priorities they face (including other upcoming donor-funded projects).*** | 🗷 |
|  | Personnel are qualified and have experience in health sector adaptation to climate change. | DrKolHero (Director, department of preventive medicine, Cambodia), and Dr Piseth Raingsey (previous director of department of preventive medicine, Cambodia, (responsible for health sector adaptation to climate change)) are both co-authors of journal articles on diarrhoeal diseases and climate change in Cambodia:   - **McIver, Lachlan J.**, Imai, Chisato, Buettner, Petra G., Gager, Paul, Chan, Vibol S., Hashizume, Masahiro, Iddings, Steven N., Kol, Hero, Raingsey, Piseth R., and Lyne, K. (2016) [*Diarrheal diseases and climate change in Cambodia: environmental epidemiology and opportunities for adaptation.*](http://researchonline.jcu.edu.au/46165/) Asia-Pacific Journal of Public Health, 28 (7). pp. 576-585 - McIver, L. J., V. S. Chan, K. J. Bowen, S. N. Iddings, K. Hero and P. P. Raingsey, 2016. Review of climate change and water-related diseases in Cambodia and findings from stakeholder knowledge assessments, *Asia Pacific Journal of Public Health,* **28**(2_suppl): 49S-58S.   Dr Hero was involved in the preparation of the Strategic Pilot Program for Climate Resilience Project, in 2011 (PSPPCRP, 2011).  ***These two people are examples of national level personnel with demonstrated experience in health sector adaptation to climate change.*** | 🗹 |
|  | Personnel are qualified and have experience in project/program management. | [#06] ***has substantial project management experience – during the interview this respondent discussed ways to encourage stakeholder ownership of APRF activities and processes, such as preparing environmental health country profiles. This project management experience is particularly important as the RFEH doesn’t provide funding for these activities***.  In 2007 Kakuko Yoshida-Nagatani (Member of Secretariat (RFEH, 2016a)) was consulted for advice on developing project management skills (Dagmar Timmer *et al.*, 2007).  ***This is evidence of thes project management experience of these two aprf personnel.*** | 🗹 |
|  | Personnel participate in ongoing training programs. | “we have to do some training and capacity building, some program, related to, say, burden of disease, how to calculate, how to evaluate, how to do some epidemiological work and so forth” [#06]  Under the heading of ‘Capacity Strengthening Programme’, the Progress Report on the Regional Forum on Environment and Health (2013–2016) describes training modules run on Climate change and Health, on Health, Environment and Development, and an Occupational Health Services Study Tour (RFEH, 2016b).  ***Training for forum personnel (ie those at the national level working in environmental health fields) are described in interview data and evaluation documents*** | 🗹 |
|  | Personnel performance is appraised (formally or informally) at least annually. |  | (PI) |
|  | The organisation/forum/project has untied funding. | The individual countries carry the primary responsibility for funding for forum related activities, and there is a big variety in how detailed forum related outputs are, such as the NEHAPs. [#02]  The countries involved don’t necessarily have the finances available ‘in-house’ to conduct the forum related activities, and the secretariat has discussed working with member countries to develop proposals for funding through mechanisms such as the GEF. [#02]  ***However despite this lack of untied funding, the purpose of the RFEH is to use peer-support and peer-pressure to encourage member states to take ownership of environmental health activities themselves (including those related to climate change), rather than relying on others to bring projects to them – although working with member states to develop proposals for the GEF blurs this distinction somewhat.***  ***As countries are responsible for funding for activities and initiatives, the funding is the responsibility of national governments (both through their tax bases and through international support)*** | 🞚 |
|  | There is evidence that the organisation/forum/project includes a component exclusively focussed on health sector adaptation to climate change | **Objectives of Climate Change TWG**   - “To enhance regional knowledge management and technical support for emerging and re-emerging infectious diseases associated with climate change, ozone depletion and eco-system change through information sharing and regional cooperation; - “To strengthen capacity building of researches and scientists in understanding the complex inter-relationship between climate change, ozone depletion, ecosystem change and human health particularly infectious diseases; and - “To promote measures to mitigate health impact of climate change using success story of decreasing of substance related to ozone depletion” (RFEH, 2014, online).   ***All three objectives include responses to climate change impacts (i.e. adaptation), but also refer to ozone depletion and eco-system change.***  However, “Ozone depletion is only the title, nothing is being done on that. Ecosystem change is very small – it is almost entirely a climate change group” [#06].  ***The forum can be considered to engage in governance exclusively around adaptation and health.*** | 🗹 |
|  | External funding to the organisation/forum/project has increased over the past 5 years. | “the secretariat – one of it’s roles is to support member states in raising funds” [#02]  “These thematic working groups were led by countries on a voluntary basis. In some cases the thematic working groups worked - quite often they had meetings they managed to get funding, but in other cases the funding was not there, and maybe the countries that were interested” [#03]  “Another aspect is of course, resources. WHO and UNEP provide financial – small amount of financial resources, which are mostly used for the travel of participants from developing countries to come to these meetings, if we had more resources we could certainly do more work on policy and assessment work” [#02]  ***The APRF does not have funding for its activities, except funding for travel of some participants to meetings.*** | 🗷 |
|  | The organisation/forum/project has multiple funding sources. | “Another aspect is of course, resources. WHO and UNEP provide financial – small amount of financial resources, which are mostly used for the travel of participants from developing countries to come to these meetings, if we had more resources we could certainly do more work on policy and assessment work – that would then allow for more substantive discussions and policy discussions at these forums, so that is something that we could do, but we don’t have ther resources for” [#02].  “the other issue would be the funding of the whole forum. It’s mainly on a voluntary collaborations and the funding has not been very stable” [#03]  “Whether the regional mechanism can survive with, again there's not much money from WHO going into that, there's some, and thankfully some, probably even more from UNEP, I'm not sure. But the countries themselves have to contribute – they have to contribute a lot of their own costs. So whether this survives or not will depend on the member [states]” [C#07]  ***The aprf is reliant on funding from the organisations which make up its secretariat in order that some participants can attend the meetings. [#03]’s quote suggests that member countries make voluntary contributions to cover aprf expenses, which would constitute multiple sources. However [C#07]’s quote indicates that the member state contributions are to cover their own costs. As the information is not completely clear, and somewhat contradictory it is classified as a somewhat*** | 🞚 |
|  | The organisation/forum/project has financial reserves. | ***As per above, the APRF relies on WHO and UNEP for participant travel money – therefore the APRF does not have financial reserves.*** | 🗷 |
|  | The organisation/forum/project has sufficient technological resources (e.g. intellectual property rights, patents, copyright, software licences etc.) to carry out its climate change adaptation mandate. |  | (PI) |
| 1. Structure, systems and processes | The organisation/forum/project has a low degree of hierarchy (i.e. few hierarchical levels). |  | (PI) |
|  | The organisation/forum/project has a human resource management system that supports the shaping of organisational culture and staff recruitment, training, development and retention. |  | (PI) |
|  | The organisation/forum/project has a financial management system that is internationally recognised. | “UNEP is an Implementing Agency of the [GEF](http://www.thegef.org/) with the World Bank and the United Nations Development Programme ([UNDP](http://www.undp.org/)) and is the only [GEF](http://www.thegef.org/) Agency whose core business is the environment.” (<http://www.unep.org/dgef/AboutUNEPGEF/tabid/54444/Default.aspx>)  ***As part of the forum secretariat, UNEP has the financial management recognition of the GEF***  ***However as noted in 3.9 & 3.10 the APRF does not appear to have its own funding, but is reliant on UNEP and WHO. Therefore, the researcher concluded that the APRF probably does not have, but could develop,p a financial management system that is financially recognised if necessary.*** | 🞚 |
|  | The organisation/forum/project applies risk management principles in its decision-making processes. |  | (PI) |
|  | The organisation/forum/project has a centralised, user-friendly internal data management system. |  | (PI) |
|  | The organisation/forum/project has a user-friendly project/program management system (e.g. that supports personnel to identify, schedule and track resources etc.). |  | (PI) |
|  | There are mechanisms that support both vertical and horizontal communication within the organisation/forum/project |  | (PI) |
|  | The organisation/forum/project has internal dispute resolution protocols. |  | (PI) |
| 1. Research and collaboration capacity | The organisation/forum/project has plans and policies that support research. | Environmental Health Country Profiles project  ***The Environmental Health Country Profiles project, initiated by the forum, supports research with climate change links (the draft synthesis report of environmental health country profiles includes the term “climate change” 37 times)*** (RFEH, 2016c). | 🗹 |
|  | There organisation/ forum/project has funds allocated for research, or facilitates access to research funds. | “WHO and UNEP together have been preparing some environmental health data sheets – pulling together data that the countries can use in their plans and NEHAPS, but generally so far at the country level it’s been WHO that’s been providing more day to day support on the development of these plans. Because WHO has country offices where we don’t” [#02].  ***The APRF doesn’t have funding of its own, but it does have the facility to organise some support from WHO country offices*** | 🗹 |
|  | The organisation/forum/ project has equipment, expertise and/or resources (e.g. access to journal articles etc.) for research, or is able to facilitate access to research related resources | ***The APRF doesn’t have funding of its own, but it does have the facility to organise some support from WHO country offices***  ***Also, WHO produces research on climate change adaptation and health, and makes this publicly available. For example who has a website of climate change and health publications*** (WHO, 2017). | 🗹 |
|  | The current organisational/forum/project strategic plan (or a similar document) outlines plans for collaboration with multiple stakeholders on health sector adaptation-related initiatives. | The APRF’s Kuala Lumpur Declaration includes that the member states, “[r]esolve to strengthen our cooperation to improve inter-agency, multisectoral, bilateral, regional and international cooperation, coordination and planning through capacity-building, and also to improve the management of common and trans-boundary and cross- border issues;  Invite other countries in the region to join the Regional Forum on Environment and Health in Southeast and East Asian Countries for the common benefit of all and for the sake of learning from and assisting each other in creating a better environment to promote health for all;  Urge governments, the private sector, civil society, nongovernmental organizations, academia, occupational groups, youth and women groups, and media to be actively engaged in this process, further building ownership and commitment”(RFEH, 2013c, pp 2-3).  ***In addition to the above, the premise of the aprf is to bring together ministers and personnel from health and environment ministries*** | 🗹 |
| ***COMPONENT 2. Effectiveness of Project/Organisation/Forum Initiative – RFEH Climate Change TWG*** | | | |
| 1. Needs and goals | There is evidence that the project/program/forum initiative is filling an existing need with relation to climate change adaptation. | The climate change and health TWG has three objectives. The second of these is:  “To strengthen capacity building of researches and scientists in understanding the complex inter-relationship between climate change, ozone depletion, ecosystem change and human health particularly infectious diseases” (UNEP, 2017b: Online).  ***With reference to climate change adaptation, capacity building is a clear area of need in Southeast Asia. In Cambodia, for example,*** “[b]ut coming into implementation is very limited. Mainly Ministry of Health would not have enough resources including financial capacity and human resource capacity to translate what they have written on the paper [laws, regulations, decrees etc…] into practicality” [C#11]. ***Also see the work of authors such as*** (Bowen *et al.*, 2015; Dany *et al.*, 2015; Willems and Baumert, 2003). | 🗹 |
|  | The adaptation focus of the project/program/forum’s initiative could be considered ‘transformational’ (i.e. there are marked shifts in the way the health sector is framed and the way it operates, leading to “larger, more profound system changes”.). | ***Maybe: the objectives of the CCTWG do not appear transformational on their own, however as a whole the APRF is highlighting the links between environment and environmental change and human health. This is part of a broader effort (e.g. Watts et al., 2015), with the IPCC also reporting on links between population health and economic development (Smith et al., 2014)***  ***Highlighting health and environment links (including climate change related) could be considered transformational because it is attempting to, in the minds of policy-makers, shift thinking away from ideas of the health sector from being a cost on society to those of the health sector bringing about long term gains for society.*** | 🞚 |
|  | Climate change adaptation is a goal of the project/program/forum initiative/activity | **Objectives of Climate Change TWG**   - “To enhance regional knowledge management and technical support for emerging and re-emerging infectious diseases associated with climate change […] through information sharing and regional cooperation; - “To strengthen capacity building of researchers and scientists in understanding the complex inter-relationship between climate change […] and human health particularly infectious diseases; and - “To promote measures to mitigate health impact of climate change using success story of decreasing of substance related to ozone depletion” (RFEH, 2014, online).   ***All three of these objectives relate directly to climate change adaptation, because the focus is on the impacts of climate change and how to deal with these impacts.*** | 🗹 |
|  | The initiative/activity goals reflect the long-range impacts of climate change. | ***As above: resolving the governance issues of coordination ties in with dealing with long range impacts of climate change – establishing robust and flexible governance mechanisms that support and encourage collaborative cooperation between health and other sectors and geographies facilitates resilience to climate change impacts. Building capacity and developing strong understandings of the relationships between climate change and human health reflects the long time-scale associated with climate change impacts.*** | 🗹 |
|  | The initiative/activity’s objectives relating to climate change adaptation are specific, measurable, achievable, realistic and time-bound (SMART). | Example from draft workplan for climate change and health TWG (2013):  “Objective: Effective management of the climate change impacts for the benefit of human health.  Activity: Creation of a task force of members for the formulation of a regional project proposal on black carbon to submit toGEF6 and adaptation fund board  Timeline: 1q 2014 – 2q 2015  Facilitator: UNEP/RRCAP” (CC TWG (RFEH), 2013: 44)  ***This example shows a weak link between the activity and the objective – while the activity may contribute towards the objective, the activity is to create a task force, and there has not been a proposal, funding or project to have any impact on effective management of climate change impacts. There is a timeline and a responsible party indicated.*** | 🗷 |
|  | Member Countries were involved in developing the climate change adaptation components of the initiative/activity | “we solicit opinions from member states through the speeches of the high level officials and especially speeches of the Ministers at the meetings, so we ask them, probably before they come for the meeting, and ask them to tell us what they think the regional forum should work on. What they think the regional forum should focus on – what kind of added value that regional forum should base – so we went through every single speech of all Ministers of the all the fourteen – some of them even two speeches per country, so we went through and then we narrow it down, what are the common issues that have been discussed – and then narrow it down, narrow it down until eventually find out that most of the Ministers are talking about, for example, action not only information sharing, but also some actions. So this includes for example, especially developing countries saying it is very important for countries to develop policies based on evidence, for example”[#06]. – ***this is the APRF***  Similarly, “there was this taskforce that was about revising the whole operation. So there was a questionnaire that was sent to all the member states focal points, all agency focal points, and we then compiled it with all the […] we needed to have a lot of follow-up to get some answers. But once we got this back, as a secretariat it was compiled into a report that was then again submitted to the focal points and to the ministries for – with a few recommendations coming from the questionnaires on how to re-structure. And then this would be the main background document for discussion in the high level meeting.” [#03] ***again this is the APRF but it gives an indication of procedures.***  From the Climate Change and Health TWG held in Jakarta, December 2013: “The chair, Ms. Sri Tantri Arundhati requested to participants that they make comments on the draft Work Plan Activities [for the Climate Change and Health TWG] starting from Brunei Darussalam who requested a quick read through. Then the chair read through the Draft Activities Work Plan. According to Dr. Hassan, the activities in the work plan are not just for the host country to implement but for all countries to offer support and assistance towards the TWG work plan” (CC TWG (RFEH), 2013)  ***The quotes from [#03] and [#06] give a sense of how the APRF operates overall in terms of decision-making inclusiveness. The quote from the TWG meeting shows that members countries were involved in developing the TWG work-plan.*** | 🗹 |
| 1. Scope | The initiative/activity addresses multiple climate or climate-induced vulnerabilities (e.g. vulnerability to sea-level rise, increased sea surface and air temperature, changing rainfall patterns etc.). | “from Cambodia mentioned that VBDs and water and food borne infectious diseases and impacts from extreme events are priority agenda to be listed” (CC TWG (RFEH), 2013: 17)  ***This is evidence that climate related impacts, to which a member state is vulnerable, are being discussed in the TWG.*** | 🗹 |
|  | The initiative/activity addresses multiple non-climate-induced vulnerabilities (e.g. poverty, deforestation etc.). | “2) Training for health and environment officials on proposal formulation to  access funding opportunities [***funding related vulnerability***] Training for health and environment personnel on climate change resilience” [***capacity vulnerability***] 3)CC&H country profiling; compilation, analysis, verification and publication of the country data acquired during the TWG meeting [***information related vulnerability***] ” (CC TWG (RFEH), 2013: 44)  “She lastly mentioned the challenges that Cambodia is facing are lack of human and financial resources and technical support.” (CC TWG (RFEH), 2013: 17)  ***The TWG has discussed a variety of areas for reducing vulnerabilities that are not directly related to climate change.*** | 🗹 |
| 1. Logic, design and adequacy | The logic/design of the initiative/activity’s climate change adaptation components is evidence-based and contextualised. | ***The objectives specified in the draft work-plan, and activities to achieve them, are derived from input from the member country representatives, using evidence they have presented from the member countries.*** | 🗹 |
|  | There is evidence that the logic/design of the initiative/activity’s climate change adaptation components is an effective means to achieve its objectives. | ***Weak links between the CCTWG’s objectives and the activities to achieve them, combined with a lack of monitoring and evaluation mean there is a lack of evidence that the design of the adaptation components is an “effective means to achieve objectives”.*** | 🗷 |
| 1. Resources | Personnel are assigned exclusively to the initiative/activity. | ***Refer to APRF resources – the secretariat does not have full-time staff therefore it cannot supply people exclusively to the CCTWG.***  ***Refer to APRF resources – resource constrained developing countries with a large number of development priorities are unlikely to be able to assign personnel exclusively to the CCTWG***  ***Also,*** the CCTWG has not met since December 2013 [#06] | 🗷 |
|  | Personnel involved with implementing the initiative/activity’s adaptation components have qualifications and experience in climate change adaptation. | Focal point for Climate Change and Health in Cambodia has been the focal point since 2009 [#09], and is the director of the preventive medicine department, which is responsible for climate change and health in Cambodia. This individual is responsible for climate change and health TWG related activities in Cambodia  “the seeds of putting that in motion came out of the TWG on climate change meeting in Jakarta. Because Dr Piseth Raingsey was there, her equivalent from the ministry of environment, climate change officer, I think on communication, the donors were there, there [were] side meetings, and including our experts from headquarters, who really do know their business on climate change and health” [C#07]  ***As noted in response to APRF personnel with experience in adaptation, Dr Piseth Rainsey has co-authored academic articles on diarrhoeal disease and climate change in Cambodia. Additionally, the CCTWG meeting in Jakarta (in Dec 2013) had climate change and health experts involved.*** | 🗹 |
|  | Personnel involved with implementing the initiative/ activity’s adaptation components have qualifications and experience in project/program management. | Kol Hero Cambodia (no evidence)  Duong Danh Manh VIE (no evidence)  Kyi Lwin Oo MM (no evidence) | (NE) |
|  | There is evidence that there are sufficient personnel to achieve the objectives of the initiative/activity. |  | (PI) |
|  | There is evidence of sufficient funding for the initiative/activity’s climate change adaptation components. | Because the purpose of the Forum is to encourage ownership at the national level for environment and health concerns, the climate change and health TWG does not have activity related funding. This was highlighted by the chair and vice-chair of the Forum in 2013: “The secretariat has been requested to propose the Regional Forum work plan with regional activities and strategic areas for collaboration for the period  2014-2016. However, the implementation of the activities of the work plan is subject to availability of funds” (RFEH, 2013a: 12)  Specifically, the climate change and health TWG draft work-plan also notes that proposed objectives and activities are “subject to availability of funds” (CC TWG (RFEH), 2013: 45).  ***Evidence of sufficient funding is lacking*** | 🗷 |
| 1. Technical efficiency | There is evidence that the initiative/activity provides value for money (cost vs. outputs). | At the 2016 Ministerial Meeting in Manila a proposal to cease the climate change and health TWG was voted down, indicating that member states value this TWG. [researcher observation during forum]  ***The initiatives are led by the national governments, and must be prioritised against competing interests in development, business, security etc… But this doesn’t really tell us whether it provides value for money.***  ***Think about it in terms of regional initiatives to support health sector adaptation – there is basically no funding for the initiative itself from the forum, however the work is getting done, and so in terms of the forum itself it is providing value for money by generating enough ownership within national settings.*** | 🗹 |
| 1. Implementation | There is evidence that the initiative/activity’s climate change adaptation components have been implemented as proposed. | Objective: “Organization of a Climate Change and Health side event at the 2014 Asia Pacific Adaptation Forum” (CC TWG (RFEH), 2013: 44)  Health was included in the adaptation forum under the disaster risk reduction topic (APAN, 2014)  ***The objective of including a side event didn’t eventuate, however health and adaptation was included as part of the Asia pacific Adaptation Forum. This could be related to the CCTWG but could be related to broader global moves toward health sector adaptation*** | 🞚 |
| 1. Monitoring and evaluation | There is evidence that the initiative/activity is internally monitored and evaluated. | “… 2013 or 2014 – it was the first and maybe the only working group on climate change – thematic working group on climate change. We was so much behind in that, because the chair of that thematic working group is the Republic of Indonesia” [#06].  ***In line with this quote, the researcher has not been able to find evidence of internal monitoring and evaluation of the CCTWG.*** | 🗷 |
|  | There is evidence that the initiative/activity is externally monitored and evaluated. | ***No evidence was found of external monitoring and evaluation*** | 🗷 |
| 1. Sustainability | There is evidence of sustained outputs from the initiative/activity. | ***Given the lack of M&E and also the reported lack of work on this TWG there is no evidence of sustained outputs.*** | (NE) |
| ***COMPONENT 3. Output Effectiveness*** | | | |
| 1. Goal attainment | There is evidence in the most recent annual report or evaluation that the climate change adaptation and health-related objectives of the organisation/project/forum are being achieved. | Table below is an excerpt from a table on pages 11-15 of the *Proposed Workplan of the Regional Forum (August 2014 – 2016):*   \| Activity/Description \| Dates/ Milestones \| Outputs/Outcomes \| Point of Contact/Partners/ Resources \| \| --- \| --- \| --- \| --- \| \| **4.3 Climate Change and Health**  To strengthen national and regional programmes on climate change impacts on health. This is also to support the WHO-WPRO and WHO-SEARO Regional Framework for Action to Protect Human Health from Effects of Climate Change in the Asia-Pacific Region” \| Second half of 2015 to  early 2016 \| **Outputs:**  **TWG - Climate Change and Health**  1. Regional workshop for the training of  officers from member agencies on:  a) GEF proposal formulation and other  funding opportunities  b)vulnerability assessments for risk  mapping  c) mainstreaming NEHAPs including in  national adaptation plans.  2. Side event at the 2014 Asia Pacific  Adaptation forum in KL.  3. Mapping of institutions and  international/national agencies dealing  with related issues.  4. Presentation on TWG at ASEAN WG on  Climate Change by the TWG chair \| **Points of Contacts:**  Indonesia (Chair of the TWG)  UNEP/WHO Secretariat for technical  Support  **Partners:**  GIZ, Coordination with air quality  activities, GEF, CCAC  **Resources:**  Cost-sharing basis between Countries  Chairing of TWG- Climate Change and  Health, member countries and  Secretariat \|   From 2013 Ministerial Meeting in Kuala Lumpur, member countries of the Regional Forum:  “Agree to cooperate to develop and implement national environmental health action plans (NEHAPs), or equivalent plans, that aim to put sustainable environment and health at the centre of development, result in sustainability and improvements in environmental quality, enhance public health, and ensure the health of the future generations in the region; “ (RFEH, 2013c: 2).  From the 2013-2016 progress report: “Twelve out of 14 Regional Forum countries have produced a National Environmental Health Action Plan (NEHAP) using WHO guidance documents. The 12 countries include: Cambodia, China, Indonesia, Japan, Republic of Korea, Lao People's Democratic Republic, Malaysia, Mongolia, Philippines, Singapore, Thailand and Viet Nam” (RFEH, 2016b: 4).  Ten of the twelve countries that have produced NEHAPs also outlined their environmental health situation. Nine of these ten countries included climate change as an environmental health issue (RFEH, 2016b).  ***Health was included at the 2014 adaptation forum under the topic of disaster risk reduction***  ***Twelve of 14 member countries have produced NEHAPs, which has clear links to climate change, as identified by the goal of having NEHAPs mainstreamed, including in national adaptation plans.*** | 🗹 |
| 1. Research and knowledge management | The organisation/project/forum produces and/or publishes research that is relevant to climate change adaptation at least annually. | ***The APRF by itself appears to have only initiated Environmental Health Country Profiles, Environmental Health Data Sheets, and NEHAPs***  ***However, as the bodies that make up the secretariat, WHO and UNEP both conduct research on climate change adaptation.***  A search of the WHO global website for publications on climate change and health shows:  2015: 7 publications  2014: 3 publications  2013: 2 publications  2012: 2 publications  2011: 1 publication  2010: 2 publications  2009: 5 publications  (See: WHO (2017))  ***There are strong links between climate change adaptation and health, therefore this is taken as evidence of published research that is relevant to climate change adaptation and health.*** | 🗹 |
|  | The organisation/project /forum makes climate change adaptation-relevant research publicly available. | The publications listed above are available on the WHO website (<http://who.int/globalchange/publications/en/>).  ***Publications are publicly available through the website*** | 🗹 |
| 1. Collaboration and advocacy | There is evidence that the organisation/project/forum collaborates with multiple stakeholders to undertake climate change adaptation and health-related activities. | The Participant list from the Regional Forum held in Manila from 6-8 August 2016 includes stakeholders from 34 countries in Asia and the Pacific, as well as Observers from 16 organisations (NGOs, multilateral and bilateral development partners, coalitions), and members of the forum secretariat (RFEH, 2016a).  To revise the operation of the APRFHE, “there was a questionnaire that was sent to all the member states focal points, all agency focal points, and we then compiled it with all the recommendations and sent it back. So there was a lot of back and forth through the focal points in the agencies” [#03].  This forum included a moderated open forum on climate change and health (APRFHE, 2016b).  The Climate Change and Health TWG meeting held in December 2013 involved TWG members from 20 countries in the Asia-Pacific region (CC TWG (RFEH), 2013).  ***There were participants at the Manila Ministerial forum from many different stakeholders***  ***The TWG members represented the governments of 20 countries in the region.*** | 🗹 |
|  | The organisation/project/forum advocates for political, financial and/or other climate change support for its Member Countries in various fora at different scales. | “but we have more challenges with developing countries. You just mentioned, rightly, just now, Cambodia – one of them. So, then I said, we still need to pursue this, but rather than being seen as we are pushing, is to find mechanism where they feel that they have to do, so for example, like we inviting them for a regional meeting, to update – is a kind of informal subtle way for them to do the country profile” [#06]  ***The environmental health country profiles have links to climate change (the draft synthesis report of environmental health country profiles includes the term “climate change” 37 times) (RFEH, 2016c). The quote above indicates that the regional forum advocated for member countries to prioritise their individual environmental health country profiles.***  “we’ve discussed the idea of preparing a project proposal for the GEF [...] on climate change and health, so we’re certainly willing to support them […] the member states [on that]. We’re – the secretariat – one of it’s roles is to support member states in raising funds” [#02]  ***The forum has a role to support member states to find financing for climate change and health activities.*** | 🗹 |
| 1. Education and training | The organisation/project/forum undertakes climate change adaptation stakeholder and/or public awareness activities. | There was a climate change and health TWG meeting in Jakarta in December 2013: “The meeting of the Thematic Working Group (TWG) on Climate Change and Health of the regional forum was held from 9 to 12 December 2013. There were 29 participants (the list of participants is in Annex 1 and the meeting agenda is in Annex 2), including members of Thematic Working Group(TWG-CC) from twenty countries in the region, secretariats from the World Health Organization(WHO), United Nations Environment Programme (UNEP) and resource Persons” (CC TWG (RFEH), 2013: 1)  ***This is evidence of stakeholder activities for climate change and health.***  At the 4^th^ Ministerial Meeting of the Forum (October 08, 2016) there was a climate change and health presentation and open forum lasting a total of 1 hour and 20 minutes (APRFHE, 2016b).  ***Stakeholders were ministers and other senior government representatives from 34 Asia-Pacific nations, and observers from mulit- and bi-lateral development organisations, NGOs, and environmental health coalitions.*** | 🗹 |
|  | The organisation/project/forum develops and/or facilitates the implementation of training programs for stakeholders in issues related to climate change adaptation. | “4.1 Climate change training module  The WHO South-East Asia Region in collaboration with the WHO Western Pacific Region and the Deutsche Gesellschaft fu r Interationale Zusammenarbeit (GIZ) convened the first Biregional Training on Climate Change and Health from 26 to 30 January 2015 at the Faculty of Medicine, University Gadjah Mada, Yogyakarta, Indonesia. The training was attended by seven countries from the Western Pacific and 10 countries from the South-East Asian Region. Most participants were from Ministries of Health, with some joining from Ministries of Environment.  The main objective of this training was to strengthen the health sector's institutional capacity to understand the linkages and streamline climate change in public health programmes; and to implement and sustain health adaptation plans. In turn, this will increase the decision-making ability of policy-makers; strengthen health systems; and address health issues in the policies and programmes of other sectors” (RFEH, 2016b: 6).  ***This training module was reported as an APRF activity and indicates training for stakeholders*** | 🗹 |
| 1. Specialised advisory services | The organisation/project/forum provides specialised climate change adaptation-related advice to Member Countries and/or other stakeholders. | Via observation – sessions at regional forum on the science and practice of health sector adaptation (e.g. one on mosquitos in Thailand) (researcher’s observation)  At The 9^th^ High Level Officials Meeting of the Regional Forum there were specialised scientific presentations on the links between climate change and health (researcher’s observation)  The Synthesis Report on Environment and Health Country Profiles summaries the “environment and health advances and challenges in the 14 countries that comprise the Regional Forum on Environment and Health in East and Southeast Asian countries: […]. The report is based on country profiles submitted by the member countries in response to a questionnaire, and recent statistics from the World Health Organization (WHO) and other United Nations agencies, in the context of sustainable development and the Sustainable Development Goals (SDGs)” (RFEH, 2016c: 2), in assist with achieving the Forum Objectives:  “1. To identify and address priority environment and health issues that require regional action;  2. To facilitate dialogue, exchange of knowledge and best practices to promote sustainable development  in the areas of environment and health;  3. To develop and sustain mechanisms for collaborative action; and  4. To mobilize material, human and technical resources to support work on environment and health”  (UNEP, 2017a: Online)  ***In addition to the researcher’s observations at the Manila forum, this provision of a synthesised summary of environment and health concerns, which include climate change and health concerns, constitutes specialised advice to the member countries (which the forum is ideally placed to provide).*** | 🗹 |

Table 1

**References**

ADB, 2008. *Strategy 2020: The Long-Term Strategic Framework of the Asian Development Bank 2008–2020*, Asian Development Bank, Mandaluyong City, Philippines. Available at: <https://www.adb.org/sites/default/files/institutional-document/32121/strategy2020-print.pdf> (accessed February 13, 2015).

ADB, 2015. *ADB, Nordic Development Fund Help GMS Counter Climate Change Health Threat,* Asian Development Bank. Available at: <https://www.adb.org/news/adb-nordic-development-fund-help-gms-counter-climate-change-health-threat> (accessed September 23, 2016).

APAN, 2014. *Activity Brief: 4th Asia-Pacific Climate Change Adaptation Forum*, Asia Pacific Adaptation Network, Bangkok, Thailand. Available at: <http://www.asiapacificadapt.net/sites/default/files/resource/attach/201411_APAN%20Activity%20Brief_APAN%20Forum%202014%20KL_fnl_0.pdf> (accessed May 02, 2017).

APRFHE, 2016a. *Information Bulletin No. 2 (Provisional List of Participants, TWG Chairs, Resource Persons, Temporary Advisers, Observers and Secretariat)*, Asia-Pacific Regional Forum on Health and Environment, Manila, The Philippines.

APRFHE, 2016b. *Programme of the Fourth Ministerial Meeting (8 October 2016, Saturday, WHO Regional Office for the Western Pacific)*, Asia-Pacific Regional Forum on Health and Environment Manila, The Philippines. Available at: <http://drustage.unep.org/system/files/RFEHDocs/programme_day3_8oct_final_4.pdf> (accessed October 01, 2016).

Bowen, K. J., Miller, F. P., Dany, V. and Graham, S., 2015. The relevance of a coproductive capacity framework to climate change adaptation: investigating the health and water sectors in Cambodia, *Ecol Soc,* **20**(1): 13.

CC TWG (RFEH), 2013. *Meeting report: Meeting of the thematic working group (TWG) on climate change and health of the regional forum on environment and health*, Thematic working group (TWG) on climate change, ozone depletion and ecosystem changes, Jakarta, Indonesia. Available at: <http://www.wpro.who.int/health_environment/publications/mtgreporttwgclimatechange2013.pdf?ua=1> (accessed March 10, 2016).

Collingwood, V., 2006. Non-governmental organisations, power and legitimacy in international society, *Review of International Studies,* **32**(03): 439-454. Available at: <https://dx.doi.org/10.1017/S0260210506007108> (accessed June 03, 2017).

Dagmar Timmer, Heather Creech and Buckler, C., 2007. *Becoming a Sustainability Leader: IISD’s role in shaping the next generation of sustainable development leadership* International Institute for Sustainable Development, Winnipeg, Canada. Available at: <https://www.iisd.org/pdf/2007/sustainability_leader.pdf> (accessed May 07, 2017).

Dany, V., Bowen, K. J. and Miller, F., 2015. Assessing the institutional capacity to adapt to climate change: a case study in the Cambodian health and water sectors, *Clim Policy,* **15**(3): 388-409.

GEF, 2015a. *GEF2020: Strategy for the GEF*, Global Environment Facility, Washington DC, USA. Available at: <https://www.thegef.org/sites/default/files/publications/GEF-2020Strategies-March2015_CRA_WEB_2.pdf> (accessed September 30, 2016).

GEF, 2015b. *Instrument for the Establishment of the Restructured Global Environment Facility*, Global Environment Facility, Washington DC, USA. Available at: <https://www.thegef.org/documents/instrument-establishment-restructured-global-environment-facility> (accessed September 27, 2016).

GEF, 2016. *Project Identification Form: Building Resilience of Health Systems in Asian LDCs to Climate Change* Global Environment Facility, Washington DC, USA. Available at: <https://www.thegef.org/sites/default/files/project_documents/ID6984_Council_Notification_letter_0.pdf> (accessed September 23, 2016).

MoE Cambodia and UNDP Cambodia, 2011. *Cambodia Human Development Report 2011 (Building Resilience: The Future of Rural Livelihoods in the Face of Climate Change)*, MoE Cambodia and UNDP Cambodia, Phnom Penh, Cambodia. Available at: <http://hdr.undp.org/sites/default/files/cambodia_2011_nhdr.pdf> (accessed May 07, 2017).

OECD, 2001. *Environment, Health & Safety News*, Organisation for Economic Co-operation and Development, Paris, France. Available at: <http://www.oecd.org/env/ehs/2724157.pdf> (accessed May 07, 2017).

PSPPCRP, 2011. *Report of Inception Workshop on Preparation of a Strategic Pilot Program For Climate Resilience Project Phase I*, Preparation of a Strategic Pilot Program for Climate Resilience Project Phase I, Climate Change Department of Ministry of Environment, Phnom Penh, Cambodia. Available at: <https://ppcrcambodia.files.wordpress.com/2013/02/3-report-of-workshop-on-03-march-2011-at-cambodiana-hotel-english.pdf> (accessed May 07, 2017).

Regional Forum on Environment and Health in Southeast and East Asian Countries, 2014. *Proposed Workplan of the Regional Forum (August 2014 – 2016)*, Regional Forum on Environment and Health in Southeast and East Asian Countries, online. Available at: <http://www.wpro.who.int/rfeh/policy_documents/7_workplan_regional_forum_2014_2016.pdf> (accessed September 23, 2016).

RFEH, 2007a. *Charter of the Regional Forum on Environment and Health: Southeast and East Asian Countries - Framework for Cooperation*, World Health Organization and United Nations Environment Programme, online. Available at: <http://www2.wpro.who.int/NR/rdonlyres/E1558401-48E7-40F3-8BD5-DD41F0FBFB22/0/Charterfinal.pdf> (accessed September 29, 2016).

RFEH, 2007b. *Communication Guidelines*, Regional Forum on Environment and Health, online. Available at: <http://www.environment-health.asia/rfeh/policy_documents/3_regional_forum_communication_guidelines.pdf> (accessed September 24, 2016).

RFEH, 2007c. *Ministerial Regional Forum on Environment and Health in Southeast and East Asian Countries 8-9 August, 2007 - Background and Agenda*, Regional Forum on Environment and Health, Bangkok, Thailand. Available at: <http://www.pcd.go.th/info_serv/EnvHealth.pdf> (accessed September 24, 2016).

RFEH, 2013a. *Chair and vice-chair report on the governance, impact, partnerships and sustainable financial mechanisms of the Regional Forum on Environment and Health*, Regional Forum on Environment and Health, Kuala Lumpur, Malaysia. Available at: <http://drustage.unep.org/system/files/RFEHDocs/1_chair_and_vice_chair_report_5_aug_2014.pdf> (accessed March 10, 2016).

RFEH, 2013b. *Framework for Cooperation of the Regional Forum on Environment and Health in Southeast and East Asian Countries* World Health Organization and United Nations Environment Programme, Kuala Lumpur, Malaysia. Available at: <http://www.environment-health.asia/rfeh/policy_documents/6_framework_for_cooperation.pdf> (accessed February 19, 2016).

RFEH, 2013c. *Kuala Lumpur Declaration on Environment And Health*, Regional Forum on Environment and Health and World Health Organization and United Nations Environment Programme, Kuala Lumpur, Malaysia. Available at: <http://www.wpro.who.int/rfeh/policy_documents/5_kuala_lumpur_declaration.pdf> (accessed February 19, 2016).

RFEH, 2014. *Thematic Working Group on Climate Change, Ozone Depletion and Ecosystem Changes,* Regional Forum on Environment and Health and World Health Organization and United Nations Environment Programme. Available at: <http://www.environment-health.asia/rfeh/working_groups/climate_change/en/index.html> (accessed September 24, 2016).

RFEH, 2016a. *Information Bulletin No. 2: Provisional List of Participants, TWG Chairs, Resource Persons, Temporary Advisers, Observers and Secretariat*, Asia-Pacific Regional Forum on Health and Environment (Previously Regional Forum on Environment and Health), Manila, The Philippines.

RFEH, 2016b. *Progress Report on the Regional Forum on Environment and Health 2013–2016*, Regional Forum on Environment and Health, Manila, The Philippines. Available at: <http://drustage.unep.org/system/files/RFEHDocs/hl9_5_progress_rfeh_2013-2016_draft4_28092016.pdf> (accessed May 02, 2017).

RFEH, 2016c. *Synthesis report of Environmental Health Country Profiles*, Regional Forum on Environment and Health in Southeast and East Asian Countries, Manila, The Philippines. Available at: <http://drustage.unep.org/system/files/RFEHDocs/synthesis_report_final_1.pdf> (accessed October 08, 2016).

RFEH, n.d. *Guidance Document on the Mechanisms for Conducting Meetings*, Regional Forum on Environment and Health, online. Available at: CHECK THIS (accessed September 23, 2016).

Smith, K. R., Woodward, A., Campbell-Lendrum, D., Chadee, D., Honda, Y., Liu, Q., Olwoch, J. M., Revich, B. and Sauerborn, R., 2014. Chapter 11: Human health: Impacts, adaptation and co-benefits, In *Climate Change 2014: Impacts, Adaptation, and Vulnerability. Part A: Global and Sectoral Aspects. Contribution of Working Group II to the Fifth Assessment Report of the Intergovernmental Panel on Climate Change* (Eds, Field, C. B., Barros, V. R., Dokken, D. J., Mach, K. J., Mastrandrea, M. D., Bilir, T. E., Chatterjee, M., Ebi, K. L., Estrada, Y. O., Genova, R. C., Girma, B., Kissel, E. S., Levy, A. N., MacCracken, S., Mastrandrea, P. R. and White, L. L.) Cambridge University Press, Cambridge, UK and New York, USA, pp. 709-754.

UNEP, 2017a. *Regional Forum on Environment and Health in Southeast and East Asian Countries: About,* United Nations Environment Programme. Available at: <http://www.unep.org/asiapacific/events/reh-2016/about> (accessed February 17, 2017).

UNEP, 2017b. *Regional Forum on Environment and Health in Southeast and East Asian Countries: Thematic Areas,* UN Environment. Available at: <http://www.unep.org/asiapacific/events/reh-2016/thematic-areas> (accessed February 17, 2017).

Watts, N., Adger, W. N., Agnolucci, P., Blackstock, J., Byass, P., Cai, W., Chaytor, S., Colbourn, T., Collins, M., Cooper, A., Cox, P. M., Depledge, J., Drummond, P., Ekins, P., Galaz, V., Grace, D., Graham, H., Grubb, M., Haines, A., Hamilton, I., Hunter, A., Jiang, X., Li, M., Kelman, I., Liang, L., Lott, M., Lowe, R., Luo, Y., Mace, G., Maslin, M., Nilsson, M., Oreszczyn, T., Pye, S., Quinn, T., Svensdotter, M., Venevsky, S., Warner, K., Xu, B., Yang, J., Yin, Y., Yu, C., Zhang, Q., Gong, P., Montgomery, H. and Costello, A., 2015. Health and climate change: policy responses to protect public health, *The Lancet*. Available at: <http://dx.doi.org/10.1016/S0140-6736(15)60854-6> (accessed August 06, 2015).

WHO, 2017. *Climate change and human health: Publications,* World Health Organization. Available at: <http://who.int/globalchange/publications/en/> (accessed May 05, 2017).

Willems, S. and Baumert, K., 2003. *Institutional capacity and climate actions*, OECD Environment Directorate, International Energy Agency, Paris, France.
