# Supplementary material for: Regional organisations supporting health sector responses to climate change in Southeast Asia
Source: Global Health. 2018 Aug 3;14:80. doi: 10.1186/s12992-018-0388-z (PMC6091073; doi:10.1186/s12992-018-0388-z)
Supplement: Supplementary file 3 — Modified FAROCCCA applied to the Asian Development Bank. (DOCX 51 kb) [file 12992_2018_388_MOESM3_ESM.docx]

**Additional file 3: Modified FAROCCCA applied to the Asian Development Bank**

| **RATING SYSTEM** | |
| --- | --- |
| 🗷 | No |
| 🞚 | To some extent |
| 🗹 | Yes |
| (NE) | No evidence |
| (PI) | Perceptual indicator or indicator not rated in this paper |

| **SUB-COMPONENT** | **INDICATOR** | **EVIDENCE** | **RATING** |
| --- | --- | --- | --- |
| ***COMPONENT 1. Input Effectiveness*** | | | |
| 1. Goals | Health sector adaptation to climate change was an initial goal of the organisation/forum/project | According to its charter, “The purpose of the [Asian Development] Bank shall be to foster economic growth and co-operation in the region of Asia and the Far East (hereinafter referred to as the "region") and to contribute to the acceleration of the process of economic development of the developing member countries in the region, collectively and individually” (ADB, 1966 (updated 1994): Article 1).  ***In 1965 the ADB charter did not include health and adaptation to climate change concerns. Neither did the 1994 update.*** | 🗷 |
|  | Health sector adaptation to climate change is a current goal of the organisation/forum/project. | “ADB will also help DMCs [developing member countries] adapt to the unavoidable impacts of climate change—including those related to health” (ADB, 2008: 14)  ***Adapting to health related impacts of climate change is a current objective of the ADB*** | 🗹 |
|  | Current strategies/plans contain specific climate change adaptation objectives for the health sector. | 2005 – 2015 Support for Preventive Health System in Vietnam does not reference climate change  The ADB’s results framework for their strategy 2020 includes a number of indicators relating to health (e.g. water and sanitation, underweight children, under five mortality), and also adaptation related (percentage of operations supporting mitigation or adaptation) (ADB, 2013b), but no indicators that explicitly combine adaptation and health.  ***There are no specific adaptation and health objectives*** | 🗷 |
|  | There is no other regional organisation/forum/project with similar climate change adaptation goals for the health sector. | GEF has an adaptation to climate change strategy, which includes “**Indicator 1.2.1.1.:** Health measures introduced to respond to climate sensitive disease (type and level)” (GEF, 2011) as an output for reducing “vulnerability in development sectors”.  The APRFHE articulates that,“Governments should address the health impacts and implications of the following priority areas of environmental concern at the local, national, regional and global levels:  • Air quality  • Water supply, hygiene and sanitation  • Solid and hazardous waste  • Toxic chemicals and hazardous substances  • Climate change, ozone depletion and ecosystem changes  • Contingency planning, preparedness and response in environmental health emergencies” (RFEH, 2007, Article 3)  ***Both the GEF and the APRF have similar health related climate change adaptation goals.*** | 🗷 |
| 1. Governance and leadership | There is visionary leadership and strategic direction for the organisation/forum/project. | “The second issue is that the bank is an investment bank, so focussing on the hard core sector, like transport, energy, water – and so, the social sector, particularly health are always a little bit struggling” … “the problem is not so much the investment itself – the problem is the soft component of the health sector project. Because health – building a hospital is not building a road. A road you build it, you have to maintain it a little bit, but a hospital there is all the operation of all the hospital, which is extremely complex and requires a lot of skills and money and attention. So, we are always a little bit in problems in designing the soft component of a project” [#16]  ***This is indicative of an organisational culture that is supportive of the status quo, and is thus less likely to pursue a visionary strategic direction.***  “this is targeted at government officials – we want government officials to understand better, to know better what is going on. And then be able to advocate for themselves – so what change in behaviour do we want to see? We want to see a change in behaviour in the people who are in situations where they could be affected by climate change. But will this project directly lead to that? Not in the course of the project. But if the country identifies as a priority a need for behaviour change, then one of the pilots – we have pilots across 12 test provinces in the three countries – something there could address behaviour change at the community level – so I would say in general the answer is no, but it is a possibility that some of the components of the project could lead to small scale behaviour change” [#13]  ***This suggests that if changes were to occur that would be good, but it is not something that is actively being pursued.*** | 🗷 |
|  | There is an evaluation of organisational/forum/project performance at least annually conducted by the organisation/forum/ project. | ADB publishes annual reports, eg. ADB (2006). 2006 Annual Evaluation Report (Manila, Philippines). See ADB (2016a).  ADB Annual reports are publicly available at:  <https://www.adb.org/documents/series/adb-annual-reports>  ***There is clear evidence that the ADB reports on their performance annually.*** | 🗹 |
|  | Organisational/forum/project decision-making is done by consensus or majority vote. | “Except as otherwise expressly provided in the Agreement, all decisions of the Board shall be made by a majority of the voting power represented at the meeting” (ADB, 2003, Section 8 (Voting)). Note that the term ‘voting power’ is unclear. Clarification from ADB charter: “The total voting power of each member shall consist of the sum of its basic votes and proportional votes.  (i) The basic votes of each member shall consist of such number of votes as results from the equal distribution among all the members of twenty (20) per cent of the aggregate sum of the basic votes and proportional votes of all the members.  (ii) The number of the proportional votes of each member shall be equal to the number of shares of the capital stock of the Bank held by that member.  2. In voting in the Board of Governors, each Governor shall be entitled to cast the votes of the member he represents. Except as otherwise expressly provided in this Agreement, all matters before the Board of Governors shall be decided by a majority of the voting power represented at the meeting.” (ADB, 1966 (updated 1994), Article 33 (Voting))  Summary: each member is allocated an equal share of basic votes (20% or total votes). The remaining 80% of the votes are allocated according to the shareholding of each of the banks members.  ***Decisions are made by majority vote, despite the majority being based on share-holding.*** | 🗹 |
|  | Organisational/forum/project personnel are qualified and/or equipped to achieve the goals of the organisation/forum/project. | “I work also with a health specialist colleague here, who is more junior, she has a PhD from Havard in public health – health management – so she’s also providing technical inputs – and I also have a colleague in our social development climate change department […] she is also a health expert and she has provided a lot of technical expertise for the project” [#13]  Example: Dr Gene Peralta (involved in design of health sector resilience building project as an ADB staff member, in 2016 retired from full-time staff and works as a consultant) is recognised by the World Health Organization for her environmental health expertise (WHO, 2004).  Susann Roth is a Senior Social Development Specialist, Asian Development Bank. She has worked in public health and the cross-over between health and development since 2006 (Roth, 2017).  Ancha Srinivasan (co-author of the health sector resilience strengthening project), is the principal climate change specialist in the ADB’s Southeast Asia Department. He “holds a Ph.D. from the University of Cambridge, UK, and has about 20 years of professional experience in interdisciplinary research and management, including climate change science and policy. Prior to joining the ADB, he was a Principal Researcher and Manager of the Climate Policy Project at the Institute for Global Environmental Strategies (IGES), Japan. He has contributed to several international initiatives, including the Intergovernmental Panel on Climate Change (IPCC), Global Environmental Outlook-IV, Millennium Ecosystems Assessment, and the System for Analysis, Research and Training (START) as an author and/or reviewer. He has edited eight books, and is an author of more than 90 publications” (APAN, 2012).  ***These are three clear examples of personnel who are qualified and have experience that support the climate change and health objectives of the ADB*** | 🗹 |
|  | Organisation/forum/project personnel are required to disclose potential conflicts of interest. |  | (PI) |
|  | The organisation/forum/project has mechanisms to attract, retain and develop talent. | “ADB will emphasize maintaining and enhancing staff skills. Staff will be encouraged to undertake training programs, and enhancement of skills and qualifications will be recognized as a factor in promotion criteria. To encourage learning from other institutions, more opportunities will be provided to staff for attachments with international development organizations and research and policy think tanks. To attract, motivate, and retain highly skilled technical staff, a career stream will be developed to allow the m (on a selective basis) to progress to the highest grade levels without having to shift to general management positions” (ADB, 2014a, p. 39).  At the ADB “We have a system of recording the skills in the – we have a kind of register – autofill questionnaire – what are your strengths and weaknesses and where would you like to have more training? And then every year or twice a year we have a discussion with our boss and we decide what training which would follow in the next few months or year” [#16]  ***The ADB has policies for recruitment and retention of talented staff. Staff at the ADB also report regular opportunities for training*** | 🗹 |
|  | Leaders create a dynamic organisational/forum/project culture, making it a desirable place to work. |  | (PI) |
|  | The organisation/forum/project is perceive by stakeholders as legitimate. | “Except as otherwise expressly provided in the Agreement, all decisions of the Board shall be made by a majority of the voting power represented at the meeting” (ADB, 2003, Section 8 (Voting))  ***Majority vote provides legitimacy among member states, although:***  “The total voting power of each member shall consist of the sum of its basic votes and proportional votes.  (i) The basic votes of each member shall consist of such number of votes as results from the equal distribution among all the members of twenty (20) per cent of the aggregate sum of the basic votes and proportional votes of all the members.  (ii) The number of the proportional votes of each member shall be equal to the number of shares of the capital stock of the Bank held by that member.  2. In voting in the Board of Governors, each Governor shall be entitled to cast the votes of the member he represents. Except as otherwise expressly provided in this Agreement, all matters before the Board of Governors shall be decided by a majority of the voting power represented at the meeting.” (ADB, 1966 (updated 1994), Article 33 (Voting)).  ***Thus voting is not equal, but based on financial interest. Also, in running projects:***  “our Vietnamese partner, it is the first time we’ve worked with them, and they don’t understand the ADB procedures, and so because we work basically in one way – We’re giving you money, and you have to do it this way”  ***Autocratic approaches tend to reduce perceived legitimacy***  Some national government agencies have expressed frustration because of lack of involvement in design aspects of adaptation and health projects (researcher’s observation).  ***Overall, the ADB has legitimacy through transparency (e.g. because processes and procedures are publicly available), but some autocratic implementation methodologies have undermined that legitimacy in some cases.*** | 🞚 |
| 1. Resources | There are organisational/forum/project personnel exclusively dedicated to health sector adaptation to climate change. | ***The ADB has health specialists and climate change specialists and environment and health specialists (Susann Roth (Public Health) Ancha Srinivasan (Climate Change Specialist), Gene Peralta (Environmental Health)) but does not appear to have health sector adaptation specialists.*** | 🞚 |
|  | Personnel are qualified and have experience in health sector adaptation to climate change. | Kathryn Bowen (consultant, working for Conseil Sante on Health Sector Resilience Strengthening Project) – multiple publications on climate change and health in South East Asia:  Bowen, K. J., F. P. Miller, V. Dany and S. Graham, 2015. The relevance of a coproductive capacity framework to climate change adaptation: investigating the health and water sectors in Cambodia, *Ecology and Society,* **20**(1): 13.  Bowen, K. J., F. Miller, V. Dany, A. J. McMichael and S. Friel, 2013. Enabling environments? Insights into the policy context for climate change and health adaptation decision-making in Cambodia, *Climate and Development,* **5**(4): 277-287.  Friel, S., K. Bowen, D. Campbell-Lendrum, H. Frumkin, A. J. McMichael and K. Rasanathan, 2011. Climate change, noncommunicable diseases, and development: the relationships and common policy opportunities, *Annual review of public health,* **32**(1): 133-147.  Nguyen, Q. A., F. Miller, K. Bowen and B. Tan Sinh, 2016. Evaluating capacity for climate change adaptation in the health and water sectors in Vietnam: constraints and opportunities, *Climate and Development*: 1-16. Available at: http://dx.doi.org/10.1080/17565529.2016.1146118 (accessed February 04, 2017).  ***Via the consulting firm, Conseil-Sante, the ADB has employed personnel with qualifications and experience in health sector adaptation to climate change.*** | 🗹 |
|  | Personnel are qualified and have experience in project/program management. | Ingo Neu is technical assistance coordinator for the ADB’s “Strengthening resilience to climate change in the health sector in the greater Mekong Subregion” project.  In 2012 He was “Chief of Party of the USAID funded PREPARE project for the remaining 9 months of a three years project in Asia and Africa, leading the project technical and administrative teams in the USA as well as in the respective countries   - Supported governments to develop pandemic and multihazard preparedness & response plans of their essential service sectors (banking & finance; telecommunication; transportation; food and water; health; etc.), bringing together public and private service providers. - Developed sector plan templates that were subsequently used by all countries to develop their sectoral preparedness plans - Established multistakeholder partnership networks including public and private service providers, donors, PACOM, AFRICOM, NGOs, etc. - Organised several high-level national workshops and simulation exercises in all countries. - Organised several high-level regional (ASEAN) and bi-regional (ASEAN-EAC) workshops and simulation exercises” (Neu, 2017: Online).   ***Ingo Neu has managed at least one project of significant complexity (across several continents), organised workshops and meetings, established partnerships and networks etc….*** | 🗹 |
|  | Personnel participate in ongoing training programs. | “ADB will emphasize maintaining and enhancing staff skills. Staff will be encouraged to undertake training programs, and enhancement of skills and qualifications will be recognized as a factor in promotion criteria. To encourage learning from other institutions, more opportunities will be provided to staff for attachments with international development organizations and research and policy think tanks” (ADB, 2014a, p. 39).  At the ADB “We have a system of recording the skills in the – we have a kind of register – autofill questionnaire – what are your strengths and weaknesses and where would you like to have more training? And then every year or twice a year we have a discussion with our boss and we decide what training which would follow in the next few months or year” [#16]  ***The adb has training policies that staff indicate are implemented.*** | 🗹 |
|  | Personnel performance is appraised (formally or informally) at least annually. | The ADB has “policies and procedures for evaluating consulting firms’ and individual consultants’ performance” and these note that evaluations are initiated "[a]t the expected date of the final report submission or at the midpoint of an assignment lasting for 12 months or more(ADB, 2014b: 1).  At the ADB “We have a system of recording the skills in the – we have a kind of register – autofill questionnaire – what are your strengths and weaknesses and where would you like to have more training? And then every year or twice a year we have a discussion with our boss and we decide what training which would follow in the next few months or year” [#16]  ***The formal policy for consultant evaluations indicates consultants and consultancy firms are evaluated at least annually.***  ***The interviewee response from an ADB staff member indicates that performance is assessed to determine future training needs.*** | 🗹 |
|  | The organisation/forum/project has untied funding. | “Thanks to robust partnerships with its development partners, ADB achieved $22.9 billion in operations in 2014 by leveraging $9.2 billion in cofinancing—a record high for ADB—with $13.7 billion of its own resources” (ADB, 2016b, online).  ***The $13.7 billion of its own resources indicates untied funding.*** | 🗹 |
|  | There is evidence that the organisation/forum/ project includes a component exclusively focussed on health sector adaptation to climate change. | The “Strengthening Resilience to Climate Change in the Health Sector in the Greater Mekong Subregion” project came from the Nordic Fund, with some funding from counterparts/national governments (ADB, 2015c).  ***This project is exclusively focussed on health and adaptation.*** | 🗹 |
|  | External funding to the organisation/forum/project has increased over the past 5 years. | “ADB uses operating income as the key measure to manage its financial position, make financial management decisions, and monitor financial ratios and parameters” (ADB, 2015a: 2)  Operating income 2011 – 2015 (in Millions of US dollars)   \| 31 Dec 2015 \| 31 Dec 2014 \| 31 Dec 2013 \| 31 Dec 2012 \| 31 Dec 2011 \| \| --- \| --- \| --- \| --- \| --- \| \| 343 \| 571 \| 469 \| 465 \| 587 \|   (ADB, 2015a: 4)  ***The operating income, as the key measurement of the ADB’s financial position has fluctuated over the five years 2011-2015, ending in 2015 below its value in 2011*** | 🗷 |
|  | The organisation/forum/project has multiple funding sources. | “Of the total co-financing, $4.4 billion was obtained through partnerships with official and other concessional financing sources, including bilateral and multilateral organizations, other public agencies, foundations, and corporate social responsibility programs. Financing support from these development partners increased by 15% in the face of global fiscal constraints, from $3.8 billion in 2013” (ADB, 2016b, online).  ***There is clear evidence of multiple funding sources (co-financing)*** | 🗹 |
|  | The organisation/forum/project has financial reserves. | In the equity section of the ADB’s Condensed Balance Sheets for Ordinary Capital Resources of the 2015 Financial Report:  (ADB, 2015a)   \| Item \| Balance ($ million) \| \| --- \| --- \| \| Ordinary reserve \| 11,981 \| \| Special reserve \| 322 \| \| Loan loss reserve \| 215 \|   ***The 2015 annual report shows over 12 billion dollars across three reserve funds.*** | 🗹 |
|  | The organisation/forum/project has sufficient technological resources (e.g. intellectual property rights, patents, copyright, software licences etc.) to carry out its climate change adaptation mandate. |  | (PI) |
| 1. Structure, systems and processes | The organisation/forum/project has a low degree of hierarchy (i.e. few hierarchical levels). |  | (PI) |
|  | The organisation/forum/project has a human resource management system that supports the shaping of organisational culture and staff recruitment, training, development and retention. |  | (PI) |
|  | The organisation/forum/project has a financial management system that is internationally recognised. | ADB’s 2013 annual financial report was audited by Deloitte, using audit standards generally acceptably in the United States of America (ADB, 2013a)  ***This standard of auditing makes it clear that the adb’s financial management system is of international standard*** | 🗹 |
|  | The organisation/forum/project applies risk management principles in its decision-making processes. |  | (PI) |
|  | The organisation/forum/project has a centralised, user-friendly internal data management system. |  | (PI) |
|  | The organisation/forum/project has a user-friendly project/program management system (e.g. that supports personnel to identify, schedule and track resources etc.). |  | (PI) |
|  | There are mechanisms that support both vertical and horizontal communication within the organisation/forum/project |  | (PI) |
|  | The organisation/forum/project has internal dispute resolution protocols. |  | (PI) |
| 1. Research and collaboration capacity | The organisation/forum/project has plans and policies that support research. | “This department where I am now is the knowledge department – the SDCC department – so we do a lot of studies, research, not directly from here, but sub-contracting to consultants, universities – or when there is a TA they work with universities. And they encourage publications, because ADB would like to be a knowledge bank and not just infrastructure investment, that is the development goal” [#14]  “There is hereby established the Asian Development Bank Institute (hereinafter referred to as "the Institute") as a subsidiary body of the Asian Development Bank” (ADBI, 2004: 5).  “ADBI provides intellectual input for policy makers in ADB’s developing member countries (DMCs). It does so by conducting research with a focus on medium- to long-term development issues of strategic importance that affects the region and through capacity building and training (CBT) activities that contribute to ADB’s overarching objective of poverty reduction” (ADB, 2017a: Online)  “The objectives of ADBI are to identify effective development strategies and improve the capacity for sound development of the agencies and organizations engaged in development work in ADB’s DMCs. ADBI focuses its activities on areas where it has a strategic advantage, such as the analysis of emerging policy issues from regional as well as medium- to long-term perspectives, and the facilitation of policy dialogue among senior DMC policy makers. ADBI also seeks to enhance its visibility, impact, and accessibility through high-quality knowledge products and services. It strives to be a trusted source of insight, knowledge, and information to which policy makers, academics, and others interested in Asia’s development issues turn for guidance.” (ADB, 2017a: Online).  “To accomplish its objectives, the Institute shall have the following functions: […](b) conduct of research on development issues with strategic implications for development thinking and policy formulation, including identification and distillation of best practices, and production of learning methods and materials related thereto;” (ADBI, 2004: 6)  ***As a subsidiary body of the ADB, the ADBI statute states that research on development issues is one of its functions*** | 🗹 |
|  | There organisation/ forum/project has funds allocated for research, or facilitates access to research funds. | “This department where I am now is the knowledge department – the SDCC department – so we do a lot of studies, research, not directly from here, but sub-contracting to consultants, universities – or when there is a TA they work with universities. And they encourage publications, because ADB would like to be a knowledge bank and not just infrastructure investment, that is the development goal.” [#14]  “We do a lot of research on economics” [#13]  The web-page for the ADB climate change program on the Asia Regional Integration Center website lists 116 climate change research papers (<https://aric.adb.org/initiative/adb-climate-change-program?section=0&subsection=research>).  The ADB is in the second phase of a global research alliances project, which is directly funded by the ADB (ie not through an external grant) (ADB, 2017c)  ***Interview data is supported by data on publications as well as projects that the adb has funded.*** | 🗹 |
|  | The organisation/forum/project has equipment, expertise and/or resources (e.g. access to journal articles etc.) for research, or is able to facilitate access to research related resources | “The ADB Library supports the information needs of ADB staff, using a collection of more than 77,000 titles that include books, journals, newspapers, commercial databases, Internet-based publications, and newswires” (ADB, 2017b: Online)  ***This is clear evidence of resourcing for research*** | 🗹 |
|  | The current organisational/ forum/project strategic plan (or a similar document) outlines plans for collaboration with multiple stakeholders on health sector adaptation-related initiatives. | In their operational plan for health (2015-2020), the ADB commits to optimizing “indirect public health outcomes from ADB infrastructure projects by strengthening collaboration between health and infrastructure sectors (primarily transport [road safety], urban [healthy cities], water [water safety], sanitation and energy), as highlighted in Appendix 3’ (ADB, 2015b: 7)  They specifically highlight working with the World Health Organization (WHO): “To measure achievements toward UHC (Universal Health Coverage), ADB is collaborating with WHO on developing monitoring frameworks with performance indicators derived from existing health information systems. These are being developed both regionally and for several countries” (ADB, 2015b: 9)  ***This is evidence of collaboration for improving health, and there are links to climate change and heatlh (eg. Water and sanitation), as well as because uhc is likely to build climate change resilience among impacted populations.***  In addition, documentation for stakeholder meetings for the “Strengthening Resilience to Climate Change in the Health Sector in the Greater Mekong Sub-region” show that “The project team works closely with implementing agencies in each country. In Viet Nam, the implementing agency is the Health Environment Management Agency, which is responsible for the coordination of climate change activities in the MOH. In Cambodia, the implementing agency is the Department of Preventive Medicine, which coordinates climate change-related interventions and is also the implementing agency for CDC2. In Lao PDR, the implementing agency is the Department of Hygiene and Health Promotion. Building on the CDC2 and the proposed GMS Health Security Project, the TA will foster regional knowledge sharing and dialogue on the impact of climate change on health and the harmonization of databases and surveillance systems. Cooperation with the WHO country offices in Cambodia, the Lao PDR, and Viet Nam is sought to strengthen vector surveillance and vulnerability mapping.” (Conseil-Sante, 2016: 1).  “The regional inception workshop [for the Strengthening Resilience to Climate Change in the Health Sector in the Greater Mekong Sub-region project] took place on 4-5 August 2016 in Hanoi. Attendees from relevant government agencies in Cambodia, Lao PDR, and Vietnam, as well as representatives from development partners and NGOs in the region, attended and contributed to discussions about the project's direction” (ADB, 2017d).  ***This shows clearly that for this project (as a health-sector project) is collaborating with a variety of organisations across the three countries where the project is being run*** | 🗹 |
| ***COMPONENT 2. Effectiveness of Project/Organisation/Forum Initiative – Strengthening Resilience to Climate Change in the Health Sector in the GMS*** | | | |
| 1. Needs and goals | There is evidence that the project/program/forum initiative is filling an existing need with relation to climate change adaptation. | “Climate change is recognized as a major threat to economic development in southeast Asia, which is one of the most climate at risk regions in the world, in particular, coastal and low-lying regions like the Greater Mekong Sub-region (GMS). Rapid infrastructure development including hydropower, roads and new urban areas, as well as the increased mobility of populations, is likely to have an impact on vector ecology and disease incidence. […] In the GMS, the World Health Organization (WHO) estimates that climate change will contribute to about 150,000 deaths annually. Populations are vulnerable to climate-induced health risks because of (i) changes in temperature and rainfall patterns that affect the incidence of vector-borne diseases (e.g. malaria and dengue) and also change in the geographical habitat of the disease vectors; (ii) extreme weather events that cause injuries, deaths, water contamination, and infectious and water-borne diseases; (iii) droughts and heavy rainfall that cause significant reduction in crop yield, that lead to low food security and malnutrition; and (iv) increased risk of heat waves in urban areas, along with forest fires that adversely affect air quality over broad areas and exacerbate the occurrence and intensity of respiratory diseases and heat strokes. […] Cambodia and the Lao PDR have a high burden of vector-borne diseases and in recent years more than 150,000 dengue cases were reported annually. In Cambodia, vector-borne and water-borne diseases (malaria, dengue and cholera) cause significant impact on health outcomes such as an increase in dengue cases. Viet Nam is more vulnerable to climate change due to regular flooding of low coastal areas and frequent typhoons and therefore an increased burden of vector-water and food borne diseases. Diseases related to frequent heat waves such as respiratory infections are increasing in the region. […]. Recent regional analyses show that a warmer, more variable climate will have adverse health effects, which significantly will impact on the poor, particularly women and children. Climate change will strain health resources of countries that already face public health challenges, poor infrastructure, poverty, and inequality. The urban and rural poor in the GMS have limited adaptive capacity given the existing burden of climate sensitive diseases, poverty, low educational attainment, and inadequate quality of health services. In this context, it will be necessary to identify investment priorities such as for climate resilient infrastructure and capacity building to cope and prevent the effects of climate sensitive diseases including those related to natural disasters and thereby strengthen governments' capacity for planning and implementing national climate change programs” (ADB, 2016c: Online)  ***The project data sheet highlights the major health related climate change impacts and links this government planning and implementation of climate change programs.*** | 🗹 |
|  | The adaptation focus of the project/program/forum’s initiative could be considered ‘transformational’ (i.e. there are marked shifts in the way the health sector is framed and the way it operates, leading to “larger, more profound system changes”.). | “we have these three knowledge products on climate change and health, and changing behaviour is certainly something we would like to see, but in the scope of a four year project – seeing that translated on the ground is pretty ambitious – especially knowing the rates of change that we see.  And this is targeted at government officials – we want government officials to understand better, to know better what is going on. And then be able to advocate for themselves – so what change in behaviour do we want to see? We want to see a change in behaviour in the people who are in situations where they could be affected by climate change. But will this project directly lead to that? Not in the course of the project. But if the country identifies as a priority a need for behaviour change, then one of the pilots – we have pilots across 12 test provinces in the three countries – something there could address behaviour change at the community level – so I would say in general the answer is no, but it is a possibility that some of the components of the project could lead to small scale behaviour change” [#13]  ***The project could lead to small-scale transformations in behaviour, but is not a direct objective of the project***  “we are looking at four areas in terms of transformation – transformation in terms institutions that we deal with – create new climate change institutions. Then policies – whether it is renewable energy policies, adaptation policies. Integration of DRM in you structures, infrastructures – adaptation concerns into infrastructure etc… and technologies – more updated technology transformation – LIDAR technology and other remote sensing in terms of looking at futures. And finally behaviour of the actors – so we want to transform institutions, policies, technologies and behaviour of actors whether it is the communities or whether it is government. But transformation is easy to say but difficult to achieve” [#12]  ***While transformation is a goal, it is not clear how it will be achieved.*** | 🞚 |
|  | Climate change adaptation is a goal of the project/program/forum initiative/activity | The project’s expected impact will be “Reduced vulnerability to climate-induced health risks, especially for vulnerable populations in GMS” (ADB, 2016c, online)  ***Reducing vulnerability to health risks is an adaptive measure because it is dealing with health risks as an impact of climate change*** | 🗹 |
|  | The initiative/activity goals reflect the long-range impacts of climate change. | “The direct costs to health [OF CLIMATE CHANGE] (excluding costs in health-determining sectors such as agriculture and water and sanitation) are estimated to be $2- $4 billion per year by 2030 worldwide” (ADB, 2016c, online).  ***Project documentation acknowledges long-term costs of climate change***  “The urban and rural poor in the GMS have limited adaptive capacity given the existing burden of climate sensitive diseases, poverty, low educational attainment, and inadequate quality of health services. In this context, it will be necessary to identify investment priorities such as for climate resilient infrastructure and capacity building to cope and prevent the effects of climate sensitive diseases including those related to natural disasters and thereby strengthen governments' capacity for planning and implementing national climate change programs” (ADB, 2016c: online)  ***Project documentation considers infrastructure investment, which in itself is a long-range consideration, and how to ensure climate-sensitive health impacts are considered in government planning.*** | 🗹 |
|  | The initiative/activity’s objectives relating to climate change adaptation are specific, measurable, achievable, realistic and time-bound (SMART). | Excerpt from Design and Monitoring Framework, in Technical Assistance Report: Strengthening Resilience to Climate Change in the Health Sector in the Greater Mekong Subregion (Financed by the Nordic Development Fund), page 6:   \| **Design Summary** \| **Performance Targets and Indicators with Baselines** \| **Data Sources and Reporting Mechanisms** \| **Assumptions and Risks** \| \| --- \| --- \| --- \| --- \| \| **Outputs**  1. Knowledge and understanding of the relationship between climate change and human health improved \| Revised national health adaptation plans based on completed vulnerability, impact, and adaptation assessment for each country and detailed assessments for 12 high-risk provinces in three countries by Q2 2016  Economic evaluation on cost-effectiveness of health adaptation developed for each country by Q2 2016  Developed modeling and forecasting of climate change related health effects to support early warning systems by Q4 2016  Integrated climate-related information in existing surveillance systems by Q4 2016  At least two options for investment projects and/ or programs on mitigating risks of climate change adaptation in the health sector identified and developed to prefeasibility stage by Q3 2017 \| ADB TA quarterly progress reports  Revised country-specific vulnerability maps \| **Assumption**  Data quality and availability are sufficient to develop tools and maps  **Risk**  Lack of cooperation and coordination between MOHs and relevant government agencies \| \| ***Indicators clearly link to objective. Each indicator has a timeframe. While indicators are not quantitative they are measurable.***  ***Some indicators (e.g. “Developed modeling and forecasting of climate change related health effects to support early warning systems”) should be more specific (e.g. For what diseases or circumstances)*** \| \| \| \| | 🗹 |
|  | Member Countries were involved in developing the climate change adaptation components of the initiative/activity | “ADB’s main form of engagement is to develop a country partnership strategy for five years or three years, depending on their planning cycle. For that we identify their priority and interest in projects, and then we also suggest these are the areas they should be looking at – so in some areas where we say, for example health and adaptation, or malaria is going to increase, dengue is going to increase because of increasing humidity or whatever – and they might say, well, if you have some free money you give it as a grant project but I’m not going to borrow for it. And some people might say, Well, if you can give $10 million grant then we are ready to borrow $10 million loan, which is concessional. But somebody might say, No I don’t want to take any money, this is not my priority, if you have some extra money you give it to some other project. So it all depends on the awareness of the individual and the readiness of the staff to listen to our ideas on emerging issues like climate change adaptation. So I can say frankly that all over south east asia, despite the fact that the higher level people recognise adaptation as a critical priority, but the lower level people are not yet ready to come and ask, We need this much, we need to borrow or we want to borrow” [#12]  “I wasn’t closely involved, but normally they have missions and they go around, and they collect interest from the countries and they show them draft proposal or concept note – to which the countries will agree or not, or they will provide inputs. And then they integrate them in the concept paper. And of course, internally when the TA has been approved here, then the whole process again will go back officially to the project and they will have that no objection to the TA before it can start” [#14]  “**During Project Design** Positive feedback about the proposed TA was received during initial discussions with the ministries of health. During fact-finding, a number of agencies/institutions and donor organizations working on climate change and health will be consulted to discuss scope and implementation arrangements” (ADB, 2016c: Online)  “The participating developing member countries—Cambodia, the Lao PDR, and Viet Nam—were involved in TA preparation and welcome the initiative^8^”  “^8^ TA fact-finding missions were conducted jointly with the NDF in April–May 2014. ADB will not undertake any activities in the relevant developing member country until receiving a written no-objection” (ADB, 2015c: 1)  ***The technical assistance report specifies that the developing member countries which are participating were involved in the ta preparation*** | 🗹 |
| 1. Scope | The initiative/activity addresses multiple climate or climate-induced vulnerabilities (e.g. vulnerability to sea-level rise, increased sea surface and air temperature, changing rainfall patterns etc.). | “Cambodia and the Lao PDR have a high burden of vector-borne diseases and in recent years more than 150,000 dengue cases were reported annually. In Cambodia, vector-borne and water-borne diseases (malaria, dengue and cholera) cause significant impact on health outcomes such as an increase in dengue cases. Viet Nam is more vulnerable to climate change due to regular flooding of low coastal areas and frequent typhoons and therefore an increased burden of vector-water and food borne diseases. Diseases related to frequent heat waves such as respiratory infections are increasing in the region” (ADB, 2016c, online).  ***The initiative considers climate sensitive diseases, sea-level rise, and extreme weather events (typhoons and heat waves)*** | 🗹 |
|  | The initiative/activity addresses multiple non-climate-induced vulnerabilities (e.g. poverty, deforestation etc.). | “Recent regional analyses show that a warmer, more variable climate will have adverse health effects, which significantly will impact on the poor, particularly women and children. Climate change will strain health resources of countries that already face public health challenges, poor infrastructure, poverty, and inequality. The urban and rural poor in the GMS have limited adaptive capacity given the existing burden of climate sensitive diseases, poverty, low educational attainment, and inadequate quality of health services. In this context, it will be necessary to identify investment priorities such as for climate resilient infrastructure and capacity building to cope and prevent the effects of climate sensitive diseases including those related to natural disasters and thereby strengthen governments' capacity for planning and implementing national climate change programs. Women and children are disproportionately more vulnerable than men to impacts of climate change. However, efforts to integrate gender perspectives in health adaptation programs, plans, and policies have also been limited” (ADB, 2016c, online).  ***The initiative considers impact of poverty and education levels on adaptive capacity, as well as quality of health services, and resultant necessary investment planning*** | 🗹 |
| 1. Logic, design and adequacy | The logic/design of the initiative/activity’s climate change adaptation components is evidence-based and contextualised. | “Changes in temperature and rainfall patterns are increasing the vulnerability of populations to the incidence of vector-borne diseases. A higher risk of heat waves in urban areas reduces air quality and exacerbates the occurrence of respiratory infections. The pace of urbanization and population mobility is in turn compounding these diseases.9 Strengthening the resilience of national health systems is a priority for Cambodia, the Lao PDR, and Viet Nam to achieve sustainable economic development. In the Greater Mekong Subregion (GMS), health has been identified as a priority sector within national adaptation plans” (ADB, 2015c: 2).  “Cambodia and the Lao PDR have a high burden of vector-borne diseases and in recent years more than 150,000 dengue cases were reported annually. In Cambodia, vector-borne and water-borne diseases (malaria, dengue and cholera) cause significant impact on health outcomes such as an increase in dengue cases. Viet Nam is more vulnerable to climate change due to regular flooding of low coastal areas and frequent typhoons and therefore an increased burden of vector-water and food borne diseases. Diseases related to frequent heat waves such as respiratory infections are increasing in the region” (ADB, 2016c: Online).  ***The design of the project is contextualised and evidence based in that it takes into account conditions in the three countries, and builds on previous projects in the region.*** | 🗹 |
|  | There is evidence that the logic/design of the initiative/activity’s climate change adaptation components is an effective means to achieve its objectives. | The project targets three outputs and one outcome. The outputs are:  “Improved integrated surveillance for climate change related impact  Strengthened institutional and human resource capacities to climate change adaptation in the health sector  Climate change adaptation is integrated in national and regional health operation plans” (ADB, 2016c: Online)  The targeted outcome is:  “Improved government capacity to cope with adverse health impact of climate change” (ADB, 2016c: Online).  ***Developing HR and institutional capacity is an important component of increasing adaptive capacity: e.g. “regional organisations should prioritise capacity-building for risk reduction within national governments over discrete project implementation, at least over the next few years. As one Caribbean interviewee noted, regional organisations can [and should] lead on “building capacity to reduce our [the region’s] exposure to risk” (interviews, January 2015)”*** (Robinson and Gilfillan, 2017). | 🗹 |
| 1. Resources | Personnel are assigned exclusively to the initiative/activity. | Review of the Techncial Assistance Report: Strengthening Resilience to Climate Change in the Health Sector in the GMS (ADB, 2015c) indicates that at the national level, public health experts, deputy team leaders and epidemiologists will be employed on a full-time basis (other positions are noted as intermittent, and these ones specify the number of person-months only: “Public health experts and deputy team leaders (estimated at 90 person-months; for three countries)” and “Epidemiologists (estimated at 108 person-months; for three countries).” (ADB, 2015c: 13).  ***This evidence is suggestive of personnel assigned exclusively to the initiative, it is not conclusive*** | 🞚 |
|  | Personnel involved with implementing the initiative/activity’s adaptation components have qualifications and experience in climate change adaptation. | Kathryn Bowen: “Kathryn is a Senior Research Fellow working at the nexus of global environmental change, global health and governance issues. She holds a PhD (ANU), MSc (International Health) (Humboldt & Frei Universities, Berlin) and BA/Psyc (Hons) (Newcastle)” (ANU, 2016, online)  “Kathryn consults to the WHO on climate change and health, as well as other national and international organisations. She was a researcher on a four-year study funded by AusAID, which investigated the factors that influence the development of public health adaptive capacity in the Asia Pacific. Kathryn recently completed a review of the member countries within the South East Asian Region of the WHO - focusing on climate change activities and preparations for future programs and policies” (ANU, 2016, online).  ***Dr Bowen is a project consultant (Conseil-Sante, 2016), and has adaptation and health experience and qualifications*** | 🗹 |
|  | Personnel involved with implementing the initiative/activity’s adaptation components have qualifications and experience in project/program management. | Ingo Neu worked as:  “Chief of Party  Company Name: International Medical Corps  Dates Employed Jan 2012 – Oct 2012 Employment Duration10 mos  Location: Indonesia, Philippines, Ghana, Uganda, Tanzania, Kenya  Taking over as Chief of Party of the USAID funded PREPARE project for the remaining 9 months of a three years project in Asia and Africa, leading the project technical and administrative teams in the USA as well as in the respective countries.   - Supported governments to develop pandemic and multihazard preparedness & response plans of their essential service sectors (banking & finance; telecommunication; transportation; food and water; health; etc.), bringing together public and private service providers. - Developed sector plan templates that were subsequently used by all countries to develop their sectoral preparedness plans. - Established multistakeholder partnership networks including public and private service providers, donors, PACOM, AFRICOM, NGOs, etc. - Organised several high-level national workshops and simulation exercises in all countries. - Organised several high-level regional (ASEAN) and bi-regional (ASEAN-EAC) workshops and simulation exercises.   All countries developed and tested sectoral preparedness & response plans for pandemics, which can also be adapted and used for other hazards” (Neu, 2017: Online).  ***This role has evidence of several project management components: organising workshops, establising partnership networks, support governments to developed preparedness and response plans.*** | 🗹 |
|  | There is evidence that there are sufficient personnel to achieve the objectives of the initiative/activity. | The project web-site mentions the need for 181 person-months of consultants for the TA (ADB, 2016c)  ***This indicates the project was planned to have sufficient human resources to achieve the outputs and outcomes*** | 🗹 |
|  | There is evidence of sufficient funding for the initiative/activity’s climate change adaptation components. | Funded by Nordic Development Fund US$4.36 million (ADB, 2015c).  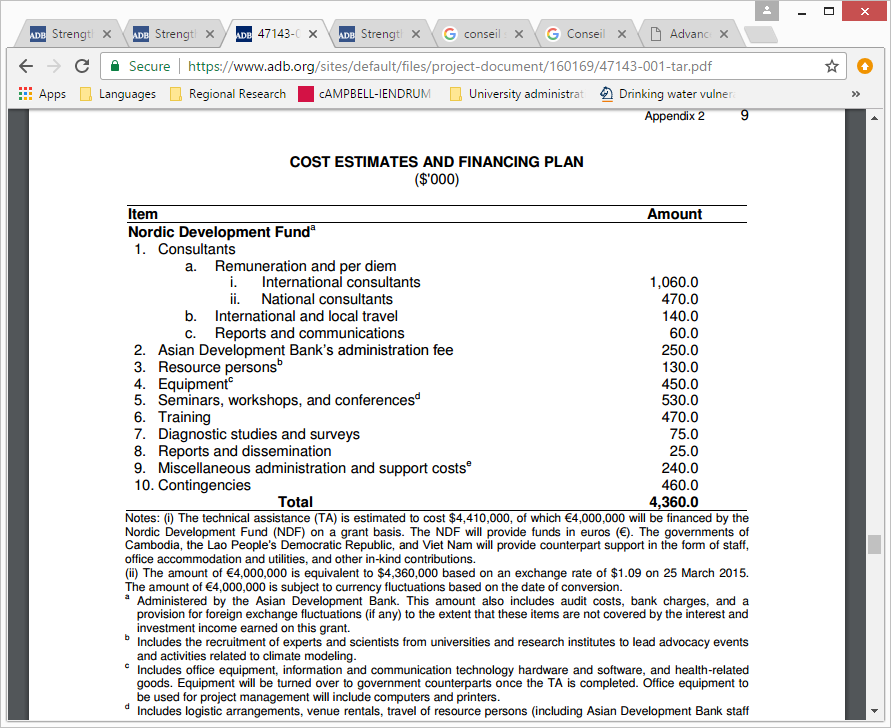  (ADB, 2015c: 9).  ***The cost estimate in the technical assistance report is broken down into the major elements including wages, equipment, events and training, administration and a contingency amount***.  ***The project budget is planned, and has been funded by the Nordic Development Fund, indicating sufficient funding.*** | 🗹 |
| 1. Technical efficiency | There is evidence that the initiative/activity provides value for money (cost vs. outputs). | “One of the things about ADB projects that is both good and bad is that there is often a lot of flexibility, TA is about building capacity, and there are a lot of different way to build capacity and some people equate it with training – so if the government needs help building its capacity in skill X we will provide training of x number of days for this number of participants, and then we will tick the box. But, I think, with this project, again it could be carried out that way, it could be a simple train them in this and train them in that and then we will have achieved our objectives. But we are looking at a more holistic way of approaching it, we are trying to build capacity for the long term and looking at the needs and the political will is as well for the capacity building. So I think the modality allows for it. But I think with somebody who just wanted to tick the boxes it could be implemented in a different way that did not build the capacity as effectively as we are planning to do” [#13]  ***This could be argued either way – this respondent is saying while it is more difficult in the short-term, to focus on capacity building including elements of political will and assessing needs will provide more value for money in the longer-term.*** | 🞚 |
| 1. Implementation | There is evidence that the initiative/activity’s climate change adaptation components have been implemented as proposed. | “Overall, despite the fact that there are delays, a lot of them are around administrative issues, because for example, VIHEMA, our Vietnamese partner, it is the first time we’ve worked with them, and they don’t understand the ADB procedures, and so because we work basically in one way – We’re giving you money, and you have to do it this way – we can see the difference between Cambodia and Laos who have worked with us before – these agencies have worked with us, so when we say, Ok, we want you to set up the advance payment facility, they say, Ok, and a week later we have all the documents. But Vietnam is like, We’re not allowed to do that. Whereas we have advance payment facilities with many other agencies in Vietnam, but they haven’t done it before, so they are not sure about the procedures and what sort of authorisations they need, and so it is a lot more hand holding, so we have to build that capacity as well to catch them up to the Cambodia and Laos agencies”  “And this is the ADB one, and it is funded, but whatever has to take place, the flag hasn't dropped, and the project hasn't [actually launched]... Usually you find out, there would be an inception workshop, something would take place. I've seen tenders are out for the individuals that will make the project work. […] $4 million over three years, but again, that's over three countries” [C#07 (September 2015)]  “The TA will be implemented from 1 August 2015 to 31 December 2018” (ADB, 2015c: 5), noting the comments from C#07 above regarding inception workshops, the inception workshop was not run until August 2016 (Conseil-Sante, 2016), 12 months later than planned.  ***Given the project inception occurred 12 months later than planned, adaptation components cannot have been implemented as planned during this 12 month period.*** | 🗷 |
| 1. Monitoring and evaluation | There is evidence that the initiative/activity is internally monitored and evaluated. | “We do an internal project evaluation at the end – it is called a TA completion report.  These completion reports are both [about accountability and looking forward] and also it depends on what the topic is – because in this kind of project we are trying to move forward the climate change and health agenda, so reflections and experience would help us understand how we need to move forward with the climate change and health pipeline” [#13]  There is no date listed for a last review mission for the project on the project website (which as last updated on 30 March 2017) (ADB, 2016c: Online).  Under the Heading “Project Outcome” on the project website there is no “Progress Towards Outcome” listed, nor was there any “Implementation Progress” listed.  ***The Quote from [#13] together with a lack of evidence on the project website indicate a lack of internal monitoring of this project.*** | 🗷 |
|  | There is evidence that the initiative/activity is externally monitored and evaluated. | “We have donor reviews, but not formally- so the donor will participate in events, and be interested in certain dimensions and they will then provide feedback, but ADB typically doesn’t have an external review, although we do have our own independent evaluation department, but they will not review every single ADB financed projects, they will pick randomly after project completion, and then evaluate the project” [#13]  ***[#13] says there are informal donor reviews, and there maybe independent evaluations by the adb’s independent evaluation department following project completion.*** | 🞚 |
| 1. Sustainability | There is evidence of sustained outputs from the initiative/activity. | ***With the lack of progress to date on this project, there is no evidence of sustained outputs*** | (NE) |
| ***COMPONENT 3. Output Effectiveness*** | | | |
| 1. Goal attainment | There is evidence in the most recent annual report or evaluation that the climate change adaptation and health-related objectives of the organisation/project/forum are being achieved. | The ADB’s 2017 Annual Review includes a review of major 2016 reports:  “Lao People’s Democratic Republic. The validation of the CPS [Country Partnership Strategy] final review covers 2012–2016. ADB’s $510 million support for lending and nonlending operations was used mostly in ANR [Agriculture, Natural resources and Rural development], education, energy, and water supply and municipal services. Support to ANR resulted in improvements in rural productivity and food security. Ongoing Greater Mekong Subregion interventions in the same sector are likely to contribute to increasing climate change resilience. ADB support also contributed to increasing access to safe water supply and sanitation; better quality secondary education and technical and vocational education and training; and electricity, including connection of poor households to the grid” (IED, 2017: 67).  ***Food security reduces health impacts such as malnutrition, and this is linked to climate change resilience in this evaluation.***  ***Increasing access to safe water and sanitation also has direct health benefits and there are clear links to climate change – sea level rise, vector- food- and water-borne diseases, which are well documented in ADB publications.***  ***Higher quality education is also linked to beneficial health outcomes as (for example) understandings of the importance of good hygiene practices become better developed.*** | 🗹 |
| 1. Research and knowledge management | The organisation/project/forum produces and/or publishes research that is relevant to climate change adaptation at least annually. | “**ADBI Library**  Although we are not a lending library for the general public and cannot satisfy individual requests for copies of our holdings, our collection is available as a bibliographic search tool for interested scholars, practitioners, and the general public. Access the ADBI library collection.  ADBI is a participating member of the collaborating network of the Japan Special Library Association.  Many ADBI publications are freely available for download.  Printed copies of ADBI publications are available through the Brookings Institution Press, our worldwide distributor, and from ADB Depository Libraries” (ADBI, 2017: Online).  A search of the ADB library for the terms “climate change” and health returned 16 items with dates (chronologically): 1998, 2008, 2009(x2), 2010, 2011(x2), 2013 (x3), 2014(x2), 2015(x2), 2017, n.d.(x1). This is of 447 items returned from a search for “climate change”. Health determining sectors return more items – eg. “climate change” and “food security” returns 12 items.  This search does not return research conducted as part of ADB projects, for example it does not return the “Climate Change Impact and Adaptation Study in The Mekong Delta – Part A (Final Report): Climate Change Vulnerability & Risk Assessment Study for Ca Mau and Kien Giang Provinces, Vietnam” (Mackay and Russell, 2011), which included a section on health in Ca Mau Province.  ***The (almost) annual publications available through the adb library, combined with additional research conducted through adb technical assistance projects indicates annual publication of adaptation and health research.*** | 🗹 |
|  | The organisation/project/forum makes climate change adaptation-relevant research publicly available. | “And it (ADB Library) is always for the public. It is easy, it is open – you can visit the library too, and ask for information, and also do research there. But if you know the title or the key words, it is out there in the public internet – it is not inside ADB website (Intranet). And then all of our monitoring reports, documents are also accessible to everyone – as long as you know the titles – you can get them” [#14]  ***The climate change and health related publications, combined with the above evidence of openly accessible library and report storage means it is publicly available.*** | 🗹 |
| 1. Collaboration and advocacy | There is evidence that the organisation/project/forum collaborates with multiple stakeholders to undertake climate change adaptation and health-related activities. | For the Health System Resilience project, “The regional inception workshop took place on 4-5 August 2016 in Hanoi. Attendees from relevant government agencies in Cambodia, Lao PDR, and Vietnam, as well as representatives from development partners and NGOs in the region, attended and contributed to discussions about the project's direction” (ADB, 2016c, online).  ***This project involved stakeholders from multiple organisations. Researcher attended the stakeholder meeting on the 3^rd^ august, and there were attendees from the country governments, academia and ngos.***  “you can look up in the ADB website – and it’s publications are – it has a lot of public information that is jointly collaborated with other NGOs and agencies” [#15]  ***Interview evidence of collaboration with other agencies to produce publications.*** | 🗹 |
|  | The organisation/project/forum advocates for political, financial and/or other climate change support for its Member Countries in various fora at different scales. | “So we are looking at four areas in terms of transformation – transformation in terms institutions that we deal with – create new climate change institutions. Then policies – whetehr it is renewable energy policies, adaptation policies. Intergration of DRM in you structures, infrastructures – adaptation concerns into infrastructure etc… and technologies – more updated technology transformation – LIDAR technology and other remote sensing in terms of looking at futures. And finally behaviour of the actors – so we want to transform institutions, policies, technologies and behaviour of actors whether it is the communities or whether it is government” [#12]  “In the Greater Mekong Subregion (GMS), health has been identified as a priority sector within national adaptation plans. Support to national health systems includes enhancing existing surveillance systems, human resource development and identifying future health infrastructure investments to help build capacity to improve tracking of disease and coordinate response at the national and regional level” (ADB, 2015c).  ***The first example shows the ADB advocating for transformations in institutions, policies, behaviours and technologies.***  ***The second example shows the ADB assisting to identify climate resilient health infrastructure investment opportunities.*** | 🗹 |
| 1. Education and training | The organisation/project/forum undertakes climate change adaptation stakeholder and/or public awareness activities. | For the Health System Resilience project, “The regional inception workshop took place on 4-5 August 2016 in Hanoi. Attendees from relevant government agencies in Cambodia, Lao PDR, and Vietnam, as well as representatives from development partners and NGOs in the region, attended and contributed to discussions about the project's direction” (ADB, 2016c, online).  ***This is an example of a climate change adaptation stakeholder activity*** | 🗹 |
|  | The organisation/project/forum develops and/or facilitates the implementation of training programs for stakeholders in issues related to climate change adaptation. | “**Output 2: Human resource skills in coping with climate change adaptation in the health sector strengthened**. The TA will support workforce development by helping to ensure the training of a new generation of competent, experienced public health staff to respond to the threats posed by climate change and incorporate mainstream climate concerns into health policies and programs. Activities include (i) development of technical guidelines for adaptation in the health sector based on existing modules, (ii) training on field epidemiology and disease surveillance related to climate change adaptation, and (iii) enhanced emergency preparedness for rapid response and recovery from extreme weather events. At the local level, community groups, including women’s groups, will need to be involved to strengthen local public health interventions, which will increase community resilience and emergency preparedness”  ***This ta (strengthening health sector resilience to climate change in the GMS) has a goal of training public health staff for health sector adaptation, with field epidemiology and disease surveillance specified as particular training areas.*** | 🗹 |
| 1. Specialised advisory services | The organisation/project/forum provides specialised climate change adaptation-related advice to Member Countries and/or other stakeholders. | “**A. International Consultants**  **Expert on climate change modeling** (estimated at 4 person-months, intermittent). The expert must have at least 8 years of experience on climate modeling and be familiar with geographic information systems. The expert will develop a regional model for examining potential health impacts from climate change, with more detailed information for the three countries covered under the TA. The model should predict health impacts of climate change at 5, 10, and 20 years for consideration in health planning and in the prioritization of adaptation actions for population health. The model should be transparent, incorporate a geographic information system, be based on open source or commonly available software, and be easily tested and updated. The expert will determine the best institutional home for public access to the model (e.g., academic or research institutes) given underlying capacities and interests, and the expert will work together in climate modeling development to build lasting capacities to support MOH interests” (ADB, 2015c).  ***There is clear evidence of specialised climate change adaptation and health-related advice specified for this ta.*** | 🗹 |

Table 1

ADB, 1966 (updated 1994). *Agreement establishing the asian development bank*, Asian Development Bank, Manila, Philippines. Available at: <https://www.adb.org/sites/default/files/institutional-document/32120/charter.pdf> (accessed October 02, 2016).

ADB, 2003. *Rules of Procedure of the Board of Governors of the Asian Development Bank*, Asian Development Bank, Manila, Philippines. Available at: <https://www.adb.org/documents/rules-procedure-board-governors-asian-development-bank> (accessed October 09, 2016).

ADB, 2008. *Strategy 2020: The Long-Term Strategic Framework of the Asian Development Bank 2008–2020*, Asian Development Bank, Mandaluyong City, Philippines. Available at: <https://www.adb.org/sites/default/files/institutional-document/32121/strategy2020-print.pdf> (accessed February 13, 2015).

ADB, 2013a. *Asian Development Bank Financial Report 2013*, Asian Development Bank, Manila, The Philippines. Available at: <https://www.adb.org/sites/default/files/institutional-document/42741/adb-financial-report-2013.pdf> (accessed October 09, 2016).

ADB, 2013b. *Results Framework 2013 - 2016: Quick Guide*, Asian Development Bank, Mandaluyong City, The Philippines. Available at: <https://www.adb.org/sites/default/files/institutional-document/33861/files/results-framework.pdf> (accessed May 26, 2017).

ADB, 2014a. *Midterm Review of Strategy 2020: Meeting the Challenges of a Transforming Asia and Pacific*, Asian Development Bank, Manila, Philippines. Available at: <https://www.adb.org/sites/default/files/institutional-document/34149/files/midterm-review-strategy-2020-r-paper.pdf> (accessed October 02,2016).

ADB, 2014b. *Project Administration Instructions: Consultant Performance Evaluation*, Asian Development Bank, Mandaluyong City, The Philippines. Available at: <https://www.adb.org/sites/default/files/institutional-document/33431/pai-2-07.pdf> (accessed May 26, 2017).

ADB, 2015a. *2015 Financial Report: Management’s Discussion and Analysis and Annual Financial Statements (31 December 2015)*, Asian Development Bank, Manila, The Philippines. Available at: <https://www.adb.org/sites/default/files/institutional-document/182852/adb-financial-report-2015.pdf> (accessed May 27, 2017).

ADB, 2015b. *Health in Asia and the Pacific: A Focused Approach to Address the Health Needs of ADB Developing Member Countries (Operational Plan for Health, 2015–2020)*, Asian Development Bank, Mandaluyong City, The Philippines. Available at: <https://www.adb.org/documents/adb-operational-plan-for-health-2015-2020> (accessed September 24, 2015).

ADB, 2015c. *Technical Assistance Report: Strengthening Resilience to Climate Change in the Health Sector in the Greater Mekong Subregion (Financed by the Nordic Development Fund)*, Asian Development Bank, Manila, The Philippines. Available at: <https://www.adb.org/sites/default/files/project-document/160169/47143-001-tar.pdf> (accessed May 02, 2017).

ADB, 2016a. *ADB Annual Reports,* Asian Development Bank. Available at: <https://www.adb.org/documents/series/adb-annual-reports> (accessed October 09, 2016).

ADB, 2016b. *Funds and Resources: Overview,* Asian Development Bank. Available at: <https://www.adb.org/site/funds/overview> (accessed October 09, 2016).

ADB, 2016c. *Regional: Strengthening Resilience to Climate Change in the Health Sector in the Greater Mekong Subregion* Asian Development Bank. Available at: <https://www.adb.org/projects/47143-001/main#project-pds> (accessed September 22, 2016).

ADB, 2017a. *About the ADB Institute,* Asian Development Bank. Available at: <https://www.adb.org/adbi/about> (accessed May 27, 2017).

ADB, 2017b. *ADB Library,* Asian Development Bank. Available at: <https://www.adb.org/about/library> (accessed May 27, 2017).

ADB, 2017c. *Regional: Establishing Global Research Alliances (Phase 2),* Asian Development Bank. Available at: <https://www.adb.org/projects/45160-001/main> (accessed May 27, 2017).

ADB, 2017d. *Regional: Strengthening Resilience to Climate Change in the Health Sector in the Greater Mekong Subregion (PDS update March 30, 2017)*, Asian Development Bank, Manila, The Philippines. Available at: <https://www.adb.org/print/projects/47143-001/main> (accessed April 25, 2017).

ADBI, 2004. *Statute of the ADB Institute*, Asian Development Bank Institute, Tokyo, Japan. Available at: <https://www.adb.org/sites/default/files/institutional-document/159300/adbi-statute-2004.pdf> (accessed May 27, 2017).

ADBI, 2017. *ADBI Library,* Asian Development Bank Institute. Available at: <https://www.adb.org/adbi/publications/library> (accessed May 04, 2017).

ANU, 2016. *Dr Kathryn Bowen* Australian National University - Research Services Division. Available at: <https://researchers.anu.edu.au/researchers/bowen-kj> (accessed October 09, 2016).

APAN, 2012. *Profile of Speakers: 2nd Asia-Pacific Climate Change Adaptation Forum (12-13 March 2012)*, Asia-Pacific Adaptation Network, Bangkok, Thailand. Available at: <http://www.asiapacificadapt.net/adaptationforum2012/sites/default/files/Profile%20of%20Resource%20Persons_3%20Feb2012.pdf> (accessed May 26, 2017).

Conseil-Sante, 2016. *CONCEPT NOTE FOR THE REGIONAL INCEPTION WORKSHOP: ADB TA-8898 REG: Regional Capacity Development for Strengthening Resilience to Climate Change in the Health Sector in the GMS Region 04 – 05 August 2016*, Conseil-Sante, Hanoi, Vietnam.

GEF, 2011. *Strategy on adaptation to climate change for the least developed countries fund (LDCF) and the special climate change fund (SCCF)*, Global Environment Facility, Washington DC, USA. Available at: <https://www.thegef.org/sites/default/files/publications/GEF-ADAPTION_STRATEGIES_2.pdf> (accessed September 30, 2016).

IED, 2017. *Annual Evaluation Review 2017*, Independent Evaluation Department - Asian Development Bank, Manila, The Philippines. Available at: <https://www.adb.org/sites/default/files/evaluation-document/214201/files/2017-aer.pdf> (accessed May 02, 2017).

Mackay, P. and Russell, M., 2011. *Socialist Republic of Viet Nam: Climate Change Impact and Adaptation Study in the Mekong Delta (Technical Assistance Report)*, Asian Development Bank, Manila, The Philippines. Available at: <https://www.adb.org/sites/default/files/project-document/73153/43295-012-tacr-03a.pdf> (accessed May 04, 2017).

Neu, I., 2017. *Ingo Neu, MD, MPH,* LinkedIn. Available at: <https://www.linkedin.com/in/ingo-neu-md-mph-63587876/?ppe=1> (accessed May 02, 2017).

RFEH, 2007. *Charter of the Regional Forum on Environment and Health: Southeast and East Asian Countries - Framework for Cooperation*, World Health Organization and United Nations Environment Programme, online. Available at: <http://www2.wpro.who.int/NR/rdonlyres/E1558401-48E7-40F3-8BD5-DD41F0FBFB22/0/Charterfinal.pdf> (accessed September 29, 2016).

Robinson, S.-a. and Gilfillan, D., 2017. Regional organisations and climate change adaptation in small island developing states, *Reg Environ Change,* **17**: 989-1004. Available at: <http://dx.doi.org/10.1007/s10113-016-0991-6> (accessed June 20, 2016).

Roth, S., 2017. *Susann Dr. Roth: Senior Social Development Specialist, Asian Development Bank and Adjunct Associate Professor at LKY SPP Singapore,* LinkedIn. Available at: <https://www.linkedin.com/in/susann-dr-roth-94029a15/?ppe=1> (accessed May 26, 2017).

WHO, 2004. *Health as a Cross-cutting Issue in dialogues on water for Food and the Environment: Report of an International Workshop*, World Health Organization, Geneva, Switzerland. Available at: <http://www.who.int/water_sanitation_health/resources/WSH04.02.pdf?ua=1> (accessed May 26, 2017).
